# Circadian KaiC Phosphorylation: A Multi-Layer Network

**DOI:** 10.1371/journal.pcbi.1000568

**Published:** 2009-11-20

**Authors:** Congxin Li, Xiaofang Chen, Pengye Wang, Weichi Wang

**Affiliations:** Laboratory of Soft Matter Physics, Beijing National Laboratory for Condensed Matter Physics, Institute of Physics, Chinese Academy of Sciences, Beijing, China; ETH Zurich, Switzerland

## Abstract

Circadian KaiC phosphorylation in cyanobacteria reconstituted *in vitro* recently initiates a series of studies experimentally and theoretically to explore its mechanism. In this paper, we report a dynamic diversity in hexameric KaiC phosphoforms using a multi-layer reaction network based on the nonequivalence of the dual phosphorylation sites (S431 and T432) in each KaiC subunit. These diverse oscillatory profiles can generate a kaleidoscopic phase modulation pattern probably responsible for the genome-wide transcription rhythms directly and/or indirectly in cyanobacteria. Particularly, our model reveals that a single KaiC hexamer is an energy-based, phosphorylation-dependent and self-regulated circadian oscillator modulated by KaiA and KaiB. We suggest that T432 is the main regulator for the oscillation amplitude, while S431 is the major phase regulator. S431 and T432 coordinately control the phosphorylation period. Robustness of the Kai network was examined by mixing samples in different phases, and varying protein concentrations and temperature. Similar results were obtained regardless of the deterministic or stochastic method employed. Therefore, the dynamic diversities and robustness of Kai oscillator make it a qualified core pacemaker that controls the cellular processes in cyanobacteria pervasively and accurately.

## Author Summary

Circadian clocks are endogenous timing mechanisms that allow living organisms to coordinate their activities with daily environmental fluctuations. In cyanobacteria, almost all the genes are rhythmically expressed with the same ∼24 h period yet exhibit a variety of phase relationships and waveforms. Remarkably, the core pacemaker ticks robustly via simple biochemical reactions carried out by three Kai proteins: KaiC undergoes circadian phosphorylation in the presence of KaiA, KaiB and ATP. In this work, we propose a reaction network modeling the Kai oscillator based on the differentiation of dual phosphorylation sites. We found a dynamic diversity in KaiC phosphorylation which may serve as a potential regulatory mechanism related to the diverse-phased genome-wide expressions in cyanobacteria. In addition, we deduce that each KaiC hexamer is a single oscillator in regulating its own phosphorylation and interactions with KaiA or/and KaiB. In complex organisms, a number of key clock components possess similar activities (e.g., phosphorylation) with multiple nonequivalent active sites, and they may also show some unusual dynamic features that are embedded in the proteins' own reaction networks. We hope our work could be helpful to study the correlations between gene expressions and circadian rhythm in prokaryotic cells, even in eukaryotic cells.

## Introduction

Cyanobacteria are the simplest organisms known that exhibit circadian rhythms. The endogenous timing system coordinates a wide range of cellular processes in cyanobacteria to the day-night cycle, including genome-wide expression [1]. For individual cyanobacterial cell, the circadian clock is autonomous with weak intercellular coupling [2],[3]. Remarkably, the core circadian clock of *S. elongatus* PCC 7942 can be reconstituted *in vitro* only with three clock proteins and ATP [4],[5]. KaiC, the central clock protein, forms a ring shaped hexamer in the presence of ATP [6],[7]. The N terminal of KaiC is essential for hexamerization, while the C terminal is responsible for catalyzing both phosphorylation and dephosphorylation [8]. Each KaiC subunit has two nonequivalent phosphorylation sites, S431 and T432 [9]–[12]. Phosphorylation or dephosphorylation occurs at the interface of two neighboring subunits [10]. The positive and negative regulations by KaiA (activator) [13] and KaiB (attenuator) [14] finally maintain a sustained circadian KaiC phosphorylation cycle *in vitro*. An ordered (or sequential) program has been proposed more recently in the rhythmic KaiC phosphorylation [11],[12]. In addition, KaiC hexamer possesses a stable ATPase activity, which may unveil the mystery pathway of energy essential for the stabilization of KaiC hexamer and the robust oscillation of KaiC phosphorylation [15].

Full understanding of the central pacemaker can provide us the insights into the mechanism of the whole cyanobacterial circadian system, e.g. the core's interaction patterns with input and output pathways. Indeed, this *in vitro* Kai oscillator has recently stimulated ever increasing theoretical elucidations and predictions. The pioneer viewpoint by Emberly and Wingreen suggests a KaiC cluster formation and monomer shuffling mechanism [16], in which the monomer shuffling is confirmed by experiments [5],[17]. A model by van Zon et al. employed the allosteric transition method to KaiC phosphorylation oscillation [18]. Many other theoretical works have been performed to mimic the KaiC phosphorylation dynamics *in vitro* or *in vivo* [12], [17], [19]–[24]. Most of the models have simplified one extremely important fact that two sites, S431 and T432 are nonequivalent in KaiC phosphorylation cycle.

In this work, we propose a step-by-step KaiC phosphorylation network based on the nonequivalence between S431 and T432 sites [9]–[12]. The phosphorylation or dephosphorylation of KaiC hexamer is designed to exhibit kinetic cooperativity, i.e. the reaction rate varies nonlinearly with KaiC own phosphorylation level (numbers of phosphorylated S431 and T432 sites). We further assume that KaiA and KaiB can strengthen the cooperativity and increase the topological complexity in the network, which culminates in an accurate and robust circadian oscillation in the Kai system. Particularly, the deterministic model shows dynamic diversities in KaiC phosphorylation cycle, e.g. a variety of phase relationships, wave forms and amplitudes. It is the reminiscence of a wide range of temporal phasing patterns exhibited in the circadian orchestration of cyanobacterial gene expression [25]. Thus, a stochastic simulation is needed to mimic the dynamic features of Kai oscillator *in vivo*. To explore the significance of the dynamic diversities, we suggest a kaleidoscopic mode that single KaiC phosphoform (hexameric) or combinations of different KaiC phosphoforms (hexameric) can act as independent oscillatory output(s) responsible for cyanobacterial gene expression in different phases. The resilience of the Kai reaction network has been well examined by varying protein concentrations, mixing samples in different phases and changing temperatures. Further examination of the model suggests that the subtle changes in KaiB-KaiC association may play a key role in explaining the slight difference between two independent studies on the KaiC dynamics with concerted increase in Kai proteins' concentrations [5],[12]. Additionally, we explore the phase response curves obtained by transient variations in KaiA concentration, and hope they could be helpful to probe the entrainment mechanism of Kai oscillator both *in vitro* and *in vivo*.

## Methods

Briefly, our method of modeling the Kai pacemaker contains two hierarchical levels. One is the construction of a Kai reaction network describing the phosphorylation, dephosphorylation of KaiC (and its complexes with KaiA and/or KaiB) and Kai protein interactions. The other level is the quantitative estimation of all the kinetic constants in the Kai reaction network based on the thermodynamic and statistical analysis of interactions within one KaiC hexamer and among Kai proteins. In the following paragraphs, we first show how an elementary KaiC phosphorylation network is built. Then we outline the main idea of the quantitative estimation for the phosphorylation (or dephosphorylation) rate constants in the network, and leave the technical details in the supporting information file Text S1. Finally, we focus on the design of the full network containing the interactions of the three Kai proteins.

### Elementary KaiC phosphorylation-dephosphorylation network

**Figure 1:**
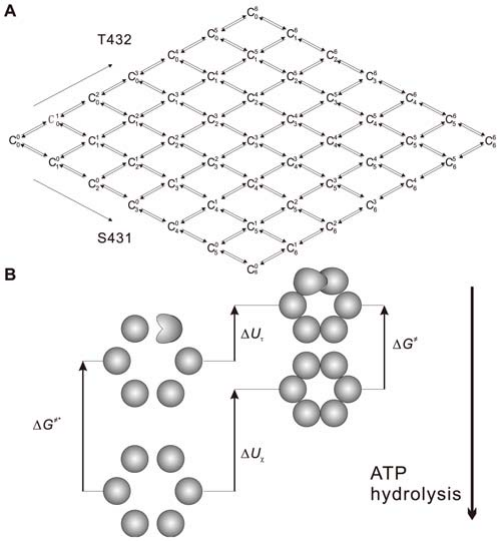
Fundamental schemes proposed in the model. (A) Reaction network for KaiC phosphorylation without KaiA and KaiB. The full multi-layer network can be found in Text S1 (section 1.1). (B) Thermodynamic box for analyzing the apparent free energy of activation in KaiC phosphorylation or dephosphorylation. On the left side, the six KaiC monomers are independent from each other. The subunit interaction that is driven by ATP hydrolysis results in the formation of stabilized and coordinated hexamers in ground state and transition state (on the right). ATP hydrolysis provides sufficient amount of extra negative free energy to make all the reactions and interactions thermodynamically possible.

Both S431 and T432 sites are indispensable for the generation of KaiC phosphorylation cycle, because mutation at either site results in total abolish of circadian phosphorylation [9],[11],[12]. As shown in Fig. 1A, a step-by-step reaction network is constructed for hexameric KaiC phosphorylation cycle (detailed reaction flows see Table S1). Each node in the network, 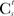, stands for a KaiC hexamer with *s* S431 and *t* T432 site(s) phosphorylated. We refer to this way of identifying the phosphorylation state of KaiC hexamer as *ST representation*. *ST representation* is convenient to outline a clear topological structure of KaiC network, although it does not explicitly contain the information of the combinations of KaiC subunits in one hexamer.

Generally, each node (or phosphoform) in the network undergoes four-directional reactions except those on the boundaries. In fact, a degenerated single site pathway or both sites considered equivalently will dramatically reduce the topological complexity of the network.

### Estimation of free energy of activation

Numerous theoretical works in protein dynamics and enzymology have provided us the insights into how the conformational changes are induced, propagated, regulated and functioned [26]–[31]. In most cases, the protein conformational fluctuations are essential for the functional regulation of molecular mechanism [32], the protein-protein interactions [33], and the chemomechanical coupling in motor proteins [34]. Definitely, information on conformational changes can be transferred to a distant site in a protein or even far in a protein cluster [35]. For Kai system, the sophisticated methods in protein dynamics enlighten us to find an approximate way on mesoscopic scale to link the changes in free energy at transition state with the rate constant for each phosphorylation or dephosphorylation step.

The Kai system is indeed driven far from equilibrium because of the continuous consumption of ATP, and most importantly, an extremely large part of ATP consumption is not attributed to KaiC kinase but to KaiC ATPase [15]. Consequently, we assume that a large portion of free energy released from ATP hydrolysis maintains the integrity and the stability of KaiC hexamer, and a relative small part of free energy is responsible for the coordination in the global structural information exchange. In Fig. 1B, we have a thermodynamic box [36]. On the left side of Fig. 1B, an isolated phosphorylation site in an isolated KaiC monomer has a quite high intrinsic free energy of activation for phosphorylation (or dephosphorylation) Δ*G* ^≠*^. Lack of interactions with other parts of the KaiC hexamer, the reaction at this site is difficult to take place and to be self-regulated. On the other hand, taking the advantage of the ATP hydrolysis, isolated KaiC monomers can form stable and coordinated hexamer at both ground state and transition state (right side in Fig. 1B). Δ*U_χ_* is the free energy difference between the isolated KaiC monomers and the stable well-coordinated hexamer at ground state, Δ*U_τ_* for the difference at transition state. Then, we have
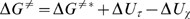
(1)


Thus, Δ*G* ^≠^ the apparent free energy of activation for a KaiC hexamer contains, in principle, all the structural information (mostly with respect to the phosphorylation states) of the six subunits.

The value of Δ*U_τ_−*Δ*U_χ_* is mainly attributed to the ATP-powered intra- and inter-subunit interactions in a KaiC hexamer, and it can reduce the intrinsic free energy of activation to increase the reaction rate provided that Δ*U_τ_−*Δ*U_χ_*<0. Furthermore, if Δ*U_τ_−*Δ*U_χ_* is sufficiently negative, the apparent free energy of activation Δ*G* ^≠^ is small enough so that the reaction rate is insensible to temperature fluctuations (according to Arrhenius equation *k* = *A*exp(-Δ*G* ^≠^/*k*_B_T)). Hence, the sufficient condition for temperature compensation must be Δ*U_τ_−*Δ*U_χ_*<0. In such case (Δ*U_χ_*>Δ*U_τ_*), within the time span of each reaction step (phosphorylation or dephosphorylation), it further suggests that more energy is required from ATP hydrolysis to maintain the stability and the coordination for ground state KaiC hexamer than that for transition state. In other words, once in a stable and well coordinated ground state, KaiC hexamer can easily overcome the energy barrier (Δ*G* ^≠^) required for phosphorylation (or dephosphorylation), because the ground state KaiC hexamer has already stood at a sufficiently high free energy level rising from ATP hydrolysis catalyzed by its own ATPase. Therefore, we postulate that the robustness against temperature fluctuation is structurally inherent in KaiC provided that the stability and the coordination of its hexamer are maintained.

In our viewpoint, the intra- and inter-subunit interactions can be generated, propagated and functioned within a KaiC hexamer, which is mostly maintained and amplified by ATP hydrolysis catalyzed by KaiC ATPase. Therefore, the phosphorylation (or dephosphorylation) rate of a KaiC hexamer is dependent on not only the local state of the phosphorylation site (or interface), but more importantly the states of adjacent subunits and even the whole hexamer. Enlightened by the sophisticated method used in protein-protein interactions [26]–[31], we roughly decompose the interaction free energy Δ*U_τ_−*Δ*U_χ_* into three hierarchical levels: local, quasi-local and global (with respect to the structure of one KaiC hexamer). To elaborate the analysis of the free energy, we use another method to describe the phosphorylation state of KaiC hexamer, the *subunit representation* C*_α,β,γ,δ._* The four subscript of C*_α,β,γ,δ_* are the numbers of m_00_ (subunit with dual non-phosphorylated S431 and T432), m_01_ (phosphorylated S431 only), m_10_ (phosphorylated T432 only) and m_11_ (dual phosphorylated S431 and T432) in one hexamer, respectively, and *α+β+γ+δ = *6. KaiC phosphorylation (or dephosphorylation) occurs at the interface of two adjacent subunits, so *subunit representation* is actually an “*interface representation*”, and m*_ij_* (*i*, *j* = 0, 1) also represents one interface in certain phosphorylation state. The rate constants and dissociation constants can be obtained by transforming the free energies from *subunit representation* into *ST representation*. All the details can be found in section 1.2 of Text S1, Table S2 and Table S3.

It should be noticed that two methods are used to describe the phosphorylation states of KaiC hexamers, i.e. *ST representation* (with 49 elements) and *subunit representation* (with 84 elements). The simplest reaction scheme for the phosphorylation (dephosphorylation) of KaiC hexamer with nonequivalent S431 and T432 sites is a network expanded by the 49 KaiC phosphoforms in *ST representation* (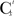), as shown in Fig. 1A. The reaction network shows a lucid dynamic relationship between S431 and T432 on KaiC hexamer. The 84 KaiC phosphorylation states in *subunit representation*, on the other hand, constitute a much more complex network, and this *subunit representation* involves so many details that hinder the analysis of dynamic reaction pathways among 84 hexameric KaiC phosphoforms. However, the *subunit representation* is more intuitive and suitable when we investigate the free energies of the intra- and inter-subunit interactions in each KaiC hexamer, whereas the *ST representation* fails in this thermo-statistical analysis, because it lacks the explicit information of phosphate distribution among six subunits. In brief, both *ST representation* and *subunit representation* have advantages and disadvantages to describe KaiC oscillator in the reaction dynamics and the estimation of statistical free energy. In this work, we take the benefits of these two *representations* and carefully perform the inter**-**transformation.

### Full Kai reaction network

In principle, the full Kai reaction network contains four layers, namely 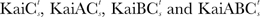. For simplicity, the four-layer network is topologically treated as two-layer network by using rapid equilibrium approximation for KaiA-KaiC and KaiA-KaiBC binding (refer to section 1.1 in Text S1). We have [LC]  =  [C] + [AC] and [LBC]  =  [BC] + [ABC].

In LC layer, KaiA binds to KaiC hexamer in a hopping fashion to stimulate the phosphorylation of KaiC. According to previous data [5], we assume that one KaiC hexamer 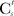 can bind one KaiA dimer forming 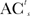, and the dissociation constant *Kat s*(KaiA dissociating from KaiC) varies mildly with KaiC phosphorylation status. Autophosphorylation of free KaiC is negligible due to its relative low rate (comparing to its dephosphorylation). All the reactions and interactions in LC layer are shown in Eq. 2.
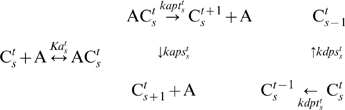
(2)


Phosphorylation state of KaiC hexamer greatly affects KaiB-KaiC association. The high-ordered association of KaiA and KaiB to one KaiC hexamer is well observed [21],[37],[38]. Here, we assign that two KaiB dimers bind to one 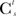 forming 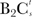. KaiB binds preferably to highly phosphorylated KaiC hexamers, especially to those with S431 phosphorylated [11]. Thus, 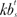, the rate constant of KaiB-KaiC association, is set to vary greatly with *s* while stay the same with *t*. Particularly, considering the weak effect of phosphorylated T432 on KaiB-KaiC association, we put 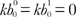, which means KaiB does not bind 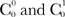 in LC layer. This assumption avoids the over early attenuation effect of KaiB on KaiA-stimulated KaiC phosphorylation, which is essential to the robustness of KaiC phosphorylation cycle against fluctuation of Kai protein concentrations. We predict that the patterns of KaiBC dephosphorylation slightly differ from those of KaiC dephosphorylation in the network. Thus, we assume S431 in KaiBC stimulates T432 dephosphorylation and attenuates its own phosphorylation. Reactions for KaiB-KaiC are represented in Eq. 3.
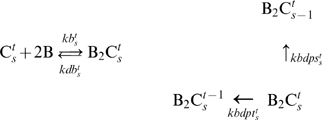
(3)


Formation of KaiABC complex is crucial to strengthen and accurately tune the interactions among the phosphorylation sites. Binary interactions such as KaiA-KaiC and KaiB-KaiC show little signs of oscillatory KaiC phosphorylation, but when three Kai proteins are mixed with ATP, self-sustained oscillation readily appears. Thus, the circadian cycling of KaiC phosphorylation attributes to the emergent property of the Kai system, and this emergent behavior definitely develops from the formation of KaiABC complex. The formation of KaiABC can be via two pathways: KaiAC + KaiB and KaiBC + KaiA. Any structural and dynamical differences between these two types of KaiABC remain to be identified. The association and dissociation of KaiA-KaiC are 15-fold and 4-fold faster than those of KaiB-KaiC, respectively [5]. Additionally, the amount of KaiBC is larger than that of KaiAC. Thus, the pathway KaiBC + KaiA is favored in this work, in which KaiABC is formed by KaiBC sequentially binding three KaiA dimers. The binding of KaiA to KaiBC is strongly dependent on KaiC phosphorylation state in contrast to the binding of KaiA to free KaiC because direct or indirect interactions may exist between KaiA and KaiB on KaiBC complex. We also assume that the interactions among these three proteins in KaiABC complex directly facilitate its own intra- and inter-subunit interactions, which generates stronger kinetic cooperativity in phosphorylation or dephosphorylation than those of other KaiC forms. Reactions for KaiABC are shown in Eq. 4, where *n* = 1, 2, 3.
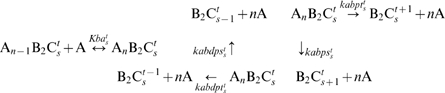
(4)


The dynamics of the full network is described by 100 equations. 98 (2×49) of them are ordinary differential equations for phosphorylation and dephosphorylation in LC and LBC layers. Other two algebraic equations represent the mass conservation law for KaiA and KaiB in the full network. Correspondingly, the total number of kinetic constants is 616 for the full Kai network (). The free energy estimation provides a way of analyzing the intrinsic correlations among those kinetic constants so that the dimension of the parameter space could be greatly reduced. Technically, the 616 kinetic constants can be automatically generated with only 88 basic parameters (Table S5). The 88 parameters are not fully independent, and constraints among which are carefully deduced by qualitative and semi-quantitative analyses of structure-based interaction free energies in one KaiC hexamer (see details in section 1.2 in Text S1).

## Results

### Functional differentiation of S431 and T432 in circadian KaiC phosphorylation

**Figure 2:**
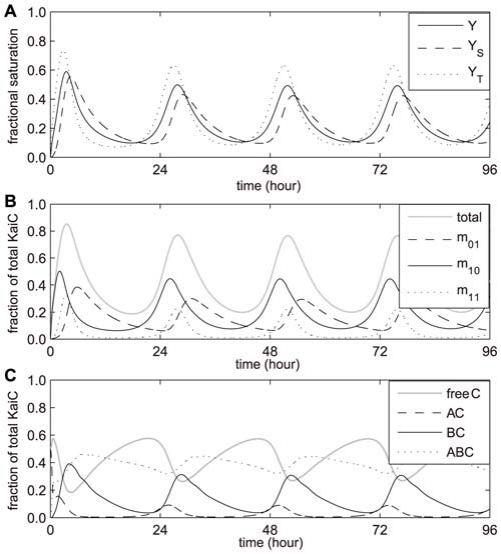
Circadian oscillations of Kai proteins in the full reaction network using deterministic method. (A) Circadian oscillation of fractional saturation of phosphates for S431 (*Y*_S_, dashed line), T432 (*Y*_T_, dotted line), and overall (*Y*, solid line). We defined 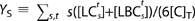, 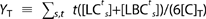 and 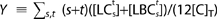, where [C]_T_ is the total amount of KaiC hexamers. (B) The circadian profiles of m_01_ (S431 phosphorylated only), m_10_ (T432 phosphorylated only), m_11_ (dual phosphorylated) and their total amount (m_01_+m_10_+m_11_). (C) Oscillatory profiles of free KaiC hexamer and its complexes. The standard protein concentrations are used: KaiA (dimer): 0.58 µM; KaiB (dimer): 1.75 µM; KaiC (hexamer): 0.58 µM.

Except where otherwise noted, results based on the deterministic method are used in this manuscript. The simulation results of circadian KaiC phosphorylation *in vitro* are shown in Fig. 2A. Here, borrowing from the concept used in multi-site substrate-enzyme binding, we define *Y*_S_, *Y*_T_ and *Y* as the fractional saturation of phosphates for S431, T432 and overall, respectively: 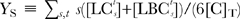, 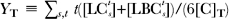 and 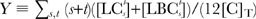, where [C]_T_ is the total amount of KaiC hexamers. *Y*_T_ is always ahead of *Y*_S_ regardless of in phosphorylation or dephosphorylation phase, and its duration is almost symmetrical between these two phases. S431 phosphorylation (*Y*_S_), on the other hand, shows an asymmetrical phase distribution and is difficult to be fully phosphorylated (the maximum number of phosphates 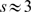). Slight changes in the strengths of interaction between S431 and T432 shows a functional differentiation of the two sites (refer to section 2.1 in Text S1). It suggests that high phosphorylation level at T432 is the main stream to determinate the amplitude in circadian KaiC phosphorylation *in vitro*, and the branch stream, S431 phosphorylation probably regulates the phase of KaiC phosphorylation cycle. T432 and S431 phosphorylation processes coordinately dominate the length of period.

It is more convenient to directly compare our results with the experiment measurements by transforming the results obtained in ST* representation* into the *subunit representation* of KaiC hexamer (refer to section 1.2.2 in Text S1). The circadian dynamics of all three monomeric KaiC phosphoforms, i.e. m_01_ (S431 phosphorylated only), m_10_ (T432 phosphorylated only) and m_11_ (dual phosphorylated), can be illustrated, as shown in Fig. 2B. The phase distribution of the three phosphoforms is m_10_ → m_11_ → m_01_, which is in well agreement with experimental results [11],[12]. The profile labeled “total” represents the sum of the three phosphoforms (m_01_+m_10_+m_11_), and it is the most usual variable characterizing the phosphorylation state of KaiC in experiments. Actually, extra variables are essential to demonstrate the mechanism of the Kai oscillator, such as m_ij_ (*i*,*j* = 0,1), *Y*_S_, *Y*_T_ and Y.

Fig. 2C shows the circadian oscillatory profiles of free KaiC and its complexes. KaiC has a high average level, and its minimum still remains ∼27% of total KaiC. KaiBC is almost anti-phase with free KaiC. The maximum amount of KaiAC only occupies ∼10.0% of total KaiC. The phases of three Kai complexes show: KaiAC the first, then KaiBC, and KaiABC the last, which have been well documented in previous experiments [5].

### Each KaiC hexamer as a single oscillator

**Figure 3:**
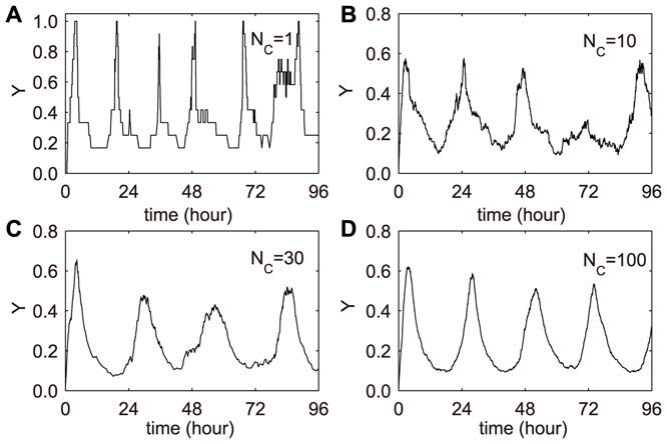
Circadian KaiC phosphorylation at low protein numbers. The ratio of the amounts of Kai proteins are fixed by N_A_ ∶ N_B_ ∶ N_C_≈1 ∶ 3 ∶ 1, where N_A_, N_B_ and N_C_ stand for the total numbers of KaiA dimers, KaiB dimers and KaiC hexamers, respectively. The ratio of total Kai protein numbers to the cell volume is set to be constant. KaiC phosphorylation cycle can be generated as N_C_ = 1 (A), N_C_ = 10 (B), N_C_ = 30 (C) and N_C_ = 100 (D).

Further, we performed a stochastic simulation of KaiC phosphorylation with low protein numbers using Gillespie algorithm [39] (see section 2.2 in Text S1). The molecule numbers of KaiA dimers, KaiB dimers and KaiC hexamers are maintained by a fixed ratio of ∼1∶3∶1 (a standard ratio of Kai proteins' concentrations in Ref. [5]). We also fixed the ratio of the Kai protein number to the cell volume. Particularly, we examined the phosphorylation cycle when there is only one KaiC hexamer (together with one KaiA dimer and three KaiB dimers), shown in Fig. 3A. It suggests that each KaiC (in the presence of KaiA and KaiB) hexamer is actually an independent oscillator with period comparable to 24 h. However, both the period and the amplitude are highly variable. Such variation can be reduced effectively by increasing the molecule numbers of Kai proteins even to a low level, shown in Fig. 3B–D.

Essentially, a single KaiC hexamer is a well coordinated subsystem with positive (KaiA) and negative (KaiB) regulators. The regulatory mechanism functions via intra- and inter-subunit interactions among the whole KaiC hexamer. Yet, the interactions within the hexamer and those from the collisions among Kai proteins (the way of the formation of Kai complexes) are accompanied with large fluctuations for one KaiC hexamer. This is why the period and amplitude are not stable for a single oscillator. However, the noise of this quasi-stable oscillator can be well reduced when we slightly increase the protein numbers.

### Node-to-node mass evolution in KaiC reaction network

**Figure 4:**
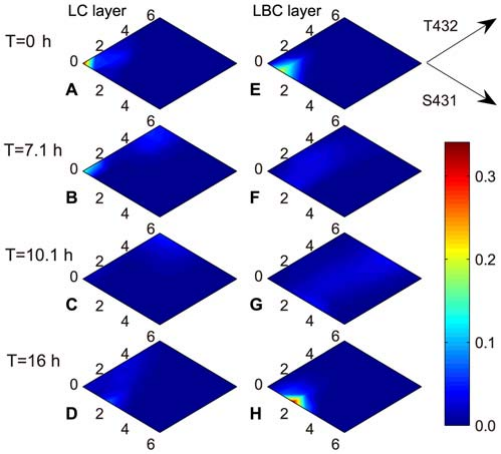
Snapshots of node-to-node mass evolution in the network. Panels A–D show the snapshots for LC layer, and panels E–H for LBC layer. The time for minimum *Y* is set to be the zero circadian time. Three other time points are chosen for comparison: T = 7.1 h (at phosphorylation phase), T = 10.1 h (the peak time) and T = 16 h (at dephosphorylation phase). The color bar shown on right corresponds to the fractions of all nodes in the contour map.

The snapshots of node-to-node mass evolution at different circadian times are shown in Fig. 4. The time for minimum *Y* is taken as the starting point of KaiC phosphorylation cycle. Interestingly, in both layers LC and LBC, no single clear-cut pathway can be identified in the whole phosphorylation cycle; instead, the reactions proceed in dispersed pathways. In effect, node-to-node mass distribution (NNMD) is found not to be synchronized between LC and LBC throughout the phosphorylation cycle. Dynamic node-to-node mass evolution in one complete cycle is shown in Video S1.

In LC layer, at initial time, the relative quantity of 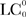 is ∼30.3% of the total amount of KaiC hexamers, while the corresponding 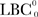 in LBC layer is merely 0.5% (Fig. 4A and 4E). From zero to ∼7.1 h, a dispersed NNMD among the low phosphorylated KaiC phosphoforms at S431 (*s*≤3) is observed in LC layer (Fig. 4B). NNMD becomes much more dispersed at the peak time (**∼**10.1 h, in Fig. 4C). Additionally, in LC layer, phosphorylation process hardly reaches the pathways 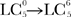 and 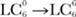. Interestingly, 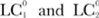 only attain their maxima even at a later time after 16 h (Fig. 4D).

LBC layer shows much different dynamic features from LC (Fig. 4E–H). The pattern of NNMD concentrates around the nodes 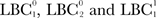 which take up ∼38.9% of the total KaiC at the initial time (Fig. 4E). Roughly, phosphorylation proceeds mainly along the pathways 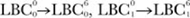 and 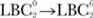 from time zero to **∼**7.1 h (Fig. 4F). Then, NNMD rapidly becomes more dispersed, and spreads almost all over the whole LBC layer at the phosphorylation peak. Notably, several nodes such as 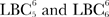 become detectable in LBC layer at the peak time, whereas their counterparts in LC layer are hardly reachable (Fig. 4G). There exists a fascinating pattern: when LC layer is absolutely in phosphorylation phase, phosphorylation and dephosphorylation are coexisted in LBC layer. Two critical nodes 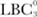 and 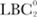 exclusively evolve into the convergent points of KaiC phosphorylation at **∼**13 h and ∼16 h, respectively (Fig. 4H).

### Dynamic diversity in Kai system

By analyzing the oscillation patterns of all the hexameric KaiC phosphoforms in the network, we found that these rhythms exhibit a variety of waveforms and phase relationships. Interestingly, it is the reminiscence of the diverse temporal phasing patterns in the genome-wide expression in cyanobacteria [25]. To examine whether the Kai oscillator has the same (or similar) dynamic diversity *in vivo*, we performed the stochastic simulation where the number of KaiC hexamers is 2000, an approximate quantity measured by experiments [14]. The stochastic simulation confirms our results, and makes a better illustration for the various waveforms.

**Figure 5:**
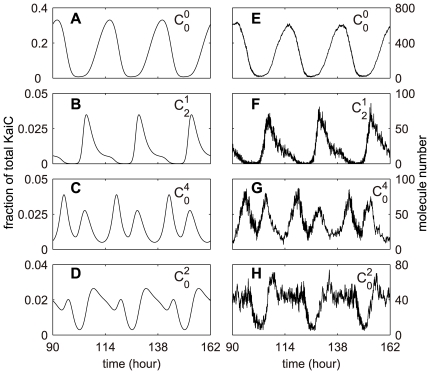
Stereotypes of diverse waveforms exhibited in KaiC phosphoforms from both deterministic and stochastic simulations. Stereotypes of four groups of waveforms of KaiC are selected: 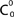 for group 1 (A and E); 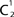 for group 2 (B and F); 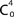 for group 3 (C and G) and 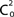 for group 4 (D and H). (A–D) for deterministic model, while (E–H) for stochastic model. The total number of KaiC hexamer is 2000 in stochastic simulation (see section 2.3 in Text S1).

The waveforms found in the KaiC phosphoforms are actually quite variable (refer to section 2.3 in Text S1), yet for convenience, we can categorize them into 4 groups. Fig. 5 shows the stereotypes of each group in both deterministic (Fig. 5A–D) and stochastic (Fig. 5E–H) simulations. Group 1 (Fig. 5A and Fig. 5E) exhibits a smooth asymmetric sinusoidal-like curve. The waveform in group 2 (Fig. 5B and Fig. 5F) shows a greatly asymmetrical “sawtooth” shape, especially in the stochastic result (Fig. 5F). Most remarkably, we found several rhythms with dual peaks (Fig. 5C and Fig. 5G) which constitute group 3. The profile in Fig. 5D appears to be dual-peak, but the sub-trough between the two peaks is not as low as that in Fig. 5C. Interestingly, the stochastic result (Fig. 5H) for the same KaiC phosphoform (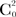) exhibits a plateau pattern. We categorize this kind of rhythms into group 4. Similar waveforms as in group 1–3 have long been observed in the oscillation profiles of bioluminescence that report the circadian gene expression patterns in cyanobacteria [25]. No pattern similar to group 4 has been found *in vivo*.

**Figure 6:**
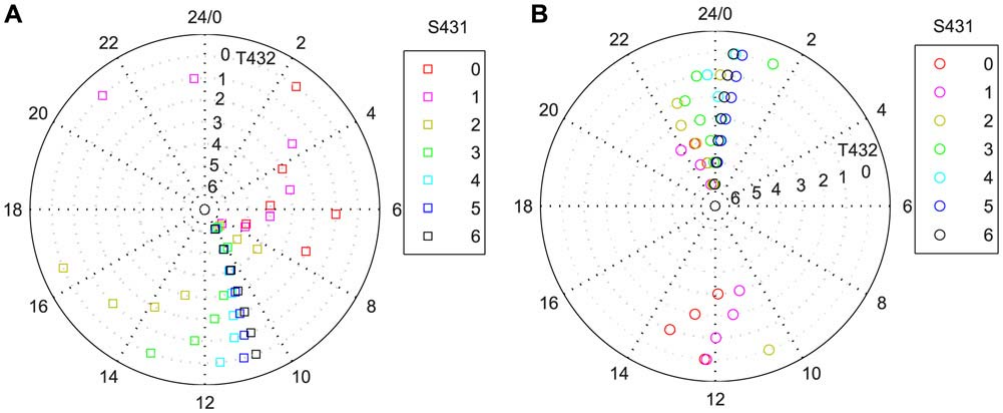
Phase distributions of hexameric KaiC phosphoforms in one circadian period. The time of minimum *Y* (the overall fractional saturation of phosphates) is taken as the zero time point of one circadian period. The sample are 49 KaiC phosphoforms defined by 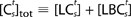. These 49 point are identified by colors (as *s*, the numbers of phosphorylated S431) and radiuses (as *t*, the numbers of phosphorylated T432) in the diagram. The phase of each phosphoforms is defined in two ways: first, by the time of maximum 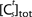 in one circadian cycle (A); the other by the time of minimum 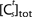 (B).

Furthermore, we analyzed the phase distribution of hexameric KaiC phosphoforms in one circadian cycle. For simplicity, we examined the phases of the 49 hexameric phosphoforms regardless of whether KaiA or/and KaiB are bound. Accordingly, the samples are 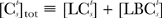, where *s*, *t* = 0,…,6. We take the trough time of *Y* (the overall fractional saturation of phosphates on KaiC) as zero time point of one circadian period. We define the phase of each KaiC phosphoform using two methods: one is to define the peak time (the highest peak for dual-peak profiles) as its phase point, while the other the time at the trough. The 49 phase points in Fig. 6A are defined by the peak time of each 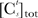. Each point is identified by both its color and the radius of its circle orbit. The color represents the number of phosphorylated S431 in one KaiC phosphoform, while the radius that of phosphorylated T432. As in Fig. 6A, the phases of KaiC phosphoforms are mainly distributed from ∼4 h to ∼16 h, during which there is a phase burst in the window between 10 h and 12 h. Cyanobacteria are a group of photoautotrophic prokaryotes, and the gene activity is much higher during the day time than at night. The phase distribution pattern of KaiC phosphoforms in Fig. 6A is well consistent with this natural habit of cyanobacteria. Interestingly, the phase points also constitute several spiral-like curves in the phase diagram (same color with variable radius). Some of them are mainly counterclockwise spiral-like curves (starting from the center), i.e. *s* = 0, 1. These points are mostly in the phosphorylation phase. When *s* = 3, 4, 5, the curves become clockwise, which correspond to the dephosphorylation phase. The curves appears to change from the counterclockwise to clockwise as *s* = 2 (the dark yellow points in Fig. 6A). It suggests that the transition from phosphorylation to dephosphorylation in the Kai system probably occurs when *s*≈2, which is consistent with our previous conclusion that S431 is the main phase regulator due to its low phase transition threshold.

Fig. 6B shows the phase distribution in which the phase of each KaiC phosphoform is defined by its own trough time. The identification of each point in the diagram is the same as in Fig. 6A (color for S431, radius for T432). Here, the phase distribution is far more converge than that in Fig. 6A. The region around 0 or 24 h is strongly favored, while that around 12 h weakly favored. Similar pattern in phase distribution has been well observed experimentally [25].

In summary, the dynamic diversity of the *in vitro* Kai system actually shares some similarities in waveforms and phase distributions with the circadian gene expression patterns in a living cyanobacterial cell. There comes a natural question: does the dynamic diversity of the central Kai oscillator contribute to the genome-wide gene expression in cyanobacteria? We do not believe the 49 KaiC phosphoforms so far contain sufficient complexity and diversity to control the ∼3000 cyanobacterial genes. One possibility is via even more complicated regulation network(s) downstream to amplify the original diversity embedded in Kai pacemaker. However, we guess there might be an alternative and more economical way for the job.

### A proposal of kaleidoscopic mode

On account of the analysis above, we propose a kaleidoscopic mechanism with stochastic fluctuation to explain the global genome regulation by Kai clock proteins. Φ the output of KaiC phosphorylation signals can be characterized as:
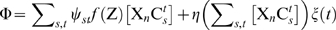
(5)


A variety of combinations (random or deterministic) of phosphorylation signals of hexameric KaiC phosphoforms would produce extremely complicated dynamic behaviors like oceans of patterns formed in a kaleidoscope. When *n* = 0 to 3, X*_n_*C*t s* denotes free KaiC, KaiAC, KaiBC and KaiABC complexes, respectively. *ψ_st_* features the transferring process of KaiC phosphorylation signal to downstream targets, and it could be a constant, a linear or a nonlinear function. *f*(Z) stands for the manners of Z, the intermediate regulators or the targets, interacting with KaiC hexamers (or Kai complexes). *ξ*(*t*) reflects Gaussian noise with zero mean, 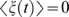. *η* represents the magnitude of the noise which is directly determined by the features of stochastic fluctuation in corresponding 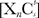.

The stochastic effect might be useful *in vivo*. Particularly, NNMD of the stochastic model shows the changes in the range and the strength of stochastic fluctuation during the phosphorylation cycle (data not shown), which may explain that two separated time points with identical average level of gene expression exhibit different fluctuations [40]. Furthermore, low molecule number in certain KaiC phosphoforms may weaken or lose their deterministic targeting to specific genes; instead, these KaiC molecules might control wider or different sets of genes due to large stochastic effect. We could hypothesize that each KaiC phosphoform may adopt one of the following modes for its phosphorylation signaling process with the time evolution in one circadian cycle: deterministic→stochastic→deterministic;

pseudo-stochastic→stochastic→pseudo-stochastic;

stochastic→stochastic→stochastic.

Bearing null phosphorylation information, 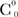 is approximately a deterministic signal with the largest amplitude and the smallest relative fluctuation (Fig. 5E). Then the whole set of KaiC phosphoforms except 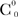 exhibits various stochastic phosphorylation signals. To the simplest case, two different output signals can be viewed in the dynamic system, one for 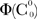 only, the other for 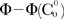.

If Φ contains a random combination of certain types of 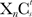, it will be a stochastic output. Different sets of random combinations result in a series of outputs, Φ*_i_* (*i* = 1, 2 …). Simply, because LC and LBC layers have never been synchronized in circadian cycle, two oscillators will then be monitored probably responsible for downstream regulation signals on gene expressions. One oscillator consists of the combination of all nodes in LC except 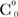, and the other in LBC. A third one is the 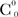 oscillator which may correlate with rhythmic chromosome compaction. There is no unique way to determine how many oscillators participate and interlock each other microscopically in the whole KaiC phosphorylation network. Hopefully, such diversified signals based on the kaleidoscopic mode and stochastic effect could be useful to harmonize the multiple oscillators [41] with the single oscillator [42] in cyanobacterial genome-wide regulations.

### Robustness in the network

**Figure 7:**
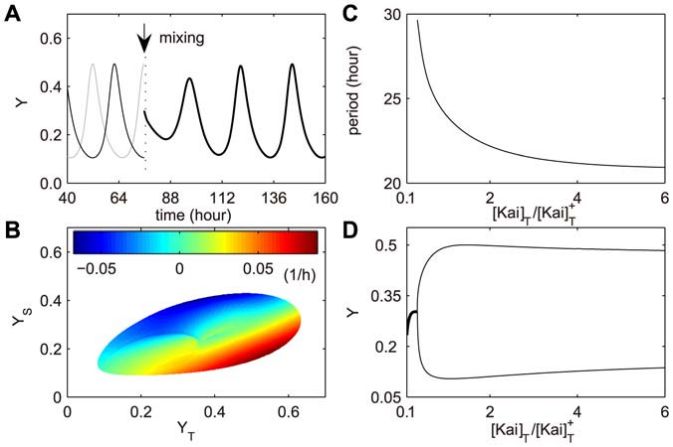
Robustness in Kai reaction network. (A) Resilience of Kai oscillator examined by mixing two anti-phased (trough and peak) samples with equal amount. The thin black and grey lines are the applied samples, and the bold black line the mixture. (B) Contour map of the initial reaction rate of *Y* after equal-amount sample mixing. The contour map is established in the interior region of the limit cycle of *Y*_S_ and *Y*_T_. (C–D) Bifurcation analysis for the period (C) and amplitude (D) of KaiC phosphorylation by varying total Kai protein concentration. 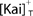 is the concentration of the standard mixture.

The synchronization of different phased Kai protein samples reveals the resilience of the Kai oscillator [43]. Mixing two samples (equal amount) with opposite phases (peak and trough) results in the dephosphorylation of the mixed KaiC system (Fig. 7A), which agrees well with recent observation [43]. Furthermore, we quantitatively analyzed how the phosphorylation at S431 and T432 contribute to the phase resetting behavior.

Fig. 7B is a contour map of the new initial reaction rate of *Y* (after equal amount mixing) versus the interior region of the limit cycle (the boundary of the phase portrait) formed by *Y*_S_ and *Y*_T_ in the phase plane. The initial values of *Y*_S_ and *Y*_T_ in the new mixture can be represented by a point in the phase plane of *Y*_S_ and *Y*_T_. Geometrically, this point is the center of the line segment connecting to the two sample points on the limit cycle, because the two samples are mixed with equal amount. If the initial reaction rate of *Y* at the new point in the phase plane is positive, the mixture goes into the phosphorylation phase, while it enters the dephosphorylation phase when the initial rate is negative. As shown in Fig. 7B, three apparent regions are displayed within the limit cycle. One fully favors the dephosphorylation phase (blue), while another the phosphorylation phase (red). The third region is the transition zone where both phosphorylation and dephosphorylation are possible. Note that approximately around the center of the phase portrait, the phosphorylation and dephosphorylation points are tangled together. A tiny perturbation can result in the system wandering between phosphorylation and dephosphorylation for a while and finally randomly entering a stable phase. All the three cases are well represented in the experiments by Ito et al. [43]. Interestingly, we found no point at which the initial rate of *Y* is zero. It suggests that the Kai oscillator is highly robust that mixing different phased samples is unable to abolish the oscillation.

The transition region can be approximately seen as a regular stripe, and the slope of which may qualitatively suggest how S431 and T432 phosphorylation contribute to the synchrony of KaiC hexamers. If the slope is parallel to the x-axis (*Y*_T_), the phase resetting is totally controlled by S431. No matter how T432 phosphorylation level varies, the mixture hardly changes its reaction direction. Likewise, if the slope of the transition region is parallel to the y-axis (*Y*_S_), the synchronization process is then fully regulated by T432 (*Y*_T_). One might imagine that when the slope's angle versus x-axis is 45°, S431 and T432 make equal contributions. Yet in our case, this angle is ∼26.5° (more likely to be attracted to *Y*_T_), thus, it suggests that S431 (*Y*_S_) is the major phase regulator for the synchrony of an ensemble of different phased KaiC hexamers. In addition, the angle of ∼26.5° corresponds to the ratio of *Y*_S_ to *Y*_T_ ∼1:2, which suggests that T432 prefers to be the main amplitude regulator.

In addition, we performed a simulation in which two equal-amount Kai samples with non-standard concentrations are mixed, keeping the final mixture to be standard. The analysis of the results may suggest a mechanism giving rise to new rhythms with periods longer than 24 hours (details can be found in section 2.8 in Text S1).

The bifurcation analysis (using XPPAUT software package [44]) as in Fig. 7C–D indicates that the oscillation is sustained even by lowering the total protein concentration to ∼0.34-fold of the standard mixture used in ref. [5]. The period changes from 29.6 h to 20.9 h as the ratio of total Kai protein goes from 0.34 to 6 (Fig. 7C), while the amplitude shows slight variation when the ratio is larger than 1∶1 (Fig. 7D). In contrast, a more recent work by Rust et al. [12] showed an opposite tendency in which the period is shortened as the protein concentrations decrease. This slight difference between the works by Kageyama et al. [5] and Rust et al. [12] needs further experimental elucidation. Furthermore, by adjusting our model's parameters (details see Table S4 and S5), we are able to obtain the same tendency of period variation (with protein concentrations) as the results by Rust et al. In the original model, we set 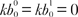, i.e. the forward reactions of KaiB binding to free 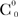 and 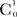 (KaiC hexamer with one T432 phosphorylated) are not allowed, whereas we let free 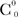 be able to bind KaiB and only leave 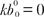 in the newly adjusted model. Based on this modification, we tune the other parameters as slightly as possible so that it can mostly reproduce the results made by Rust et al., and meanwhile keep all our earlier simulation results valid (refer to section 2.6 in Text S1).

### Examination of the model by recent experimental results

Using the new parameter set, our simulation results are consistent with the experiment of KaiC dynamics under concerted variations in Kai proteins' concentrations by Rust et al. [12]. We also reproduce some other important results given by Rust et al.

**Figure 8:**
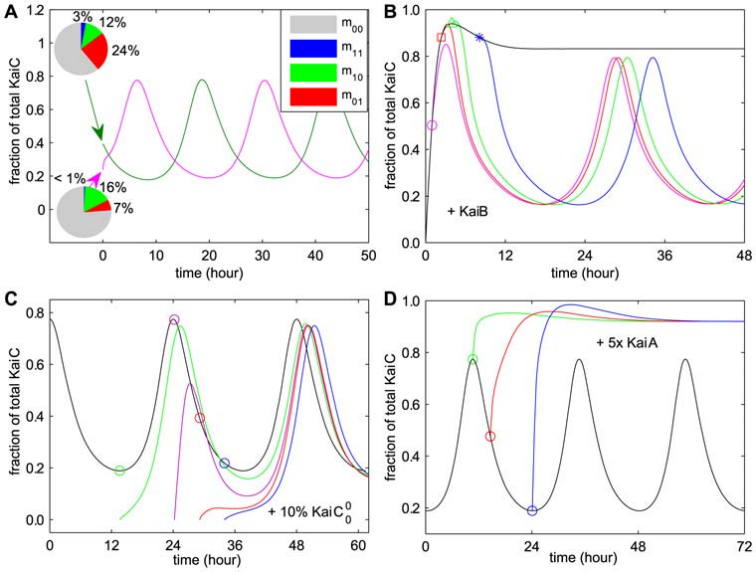
Further examination of the model by recent experimental data. (A) The distribution of KaiC phosphoforms determines the phase of KaiC phosphorylation. The KaiC sample enriched in m_10_ (monomer with T432 phosphorylated only) is obtained as follows: simulating binary KaiA and unphosphorylated KaiC reaction for 3 hours and then removing KaiA; then adding KaiB for KaiB-KaiC binary reaction for another 3 hours. The m_01_ (monomer with S431 phosphorylated only) enriched KaiC is prepared by mixing KaiA and KaiC for 20 hours, removing KaiA, and allowing KaiC dephosphorylate for 2.4 hours. (B) Standard amount of KaiB is introduced at different time points during KaiA-KaiC reaction process. (C) Extra unphosphorylated KaiC with 10% standard concentration of KaiC is introduced at various circadian time points in KaiC phosphorylation. Colored profiles (not black) show the phosphorylation dynamics for the added KaiC samples after their introductions. (D) KaiC phosphorylation profiles with 5× standard KaiA added at different circadian time points.

(i) The phase of KaiC phosphorylation is uniquely determined by the distribution of KaiC phosphoforms. Particularly, the phosphorylation state of S431 plays a more significant role in phase modulation. As shown in Fig. 8A, when the initial value of m_01_ (monomer with only S431 phosphorylated) reaches 7% of total KaiC, KaiC immediately enters the phosphorylation phase. As m_01_ takes up 24% of the total KaiC at the starting point, dephosphorylation phase is favored. The results are mostly consistent with the experimental data shown by Rust et al. Both experimental and theoretical studies suggest that S431 is more likely a phase regulator with a threshold of phosphate number approximately between 0.4 and 1.4. Below the threshold KaiC enters the phosphorylation phase, and above which KaiC prefers dephosphorylation.

(ii) Transient response curves of KaiB introduction into KaiA-KaiC reaction can be mimicked with our model, in which the explicit KaiB-KaiC and KaiA-KaiB-KaiC interactions are considered. In Fig. 8B, standard amount of KaiB is added at different time points in the binary KaiA-KaiC reaction mixture. At the early stage of KaiC phosphorylation in the presence of KaiA, KaiB has little effect on KaiC phosphorylation dynamics, whereas the attenuation of KaiB emerges significantly at a certain KaiC phosphorylation level. The attenuation effect of KaiB on KaiA mainly depends on the S431 phosphorylation level. Our model design agrees with the experimental fact that S431 is crucial for KaiB-KaiC interaction. Moreover, KaiB-KaiC formation induces competition between KaiA and KaiB binding to KaiC, and meanwhile the formation of KaiABC complex facilitates KaiB's attenuation on KaiA. The phase of S431 phosphorylation is always delayed compared with that of T432 phosphorylation (due to the differentiation of their reactivity, Fig. 2A and B). Therefore, KaiB takes effect (as an attenuator) in the late phase of KaiC phosphorylation.

(iii) The activity of KaiA is carefully examined according to the methods applied by Rust et al., i.e. to perturb the reaction system by adding small amount (10% standard KaiC) of purely unphosphorylated KaiC and introducing overdose of free KaiA at different circadian time points. Both results agree well with the corresponding experiments by Rust et al., as shown in Fig. 8C and D. During the whole phosphorylation phase, the extra 10% unphosphorylated KaiC introduced can also undergo phosphorylation stimulated by KaiA, while the activity of KaiA is greatly reduced in the dephosphorylation phase (Fig. 8C). In Fig. 8D, KaiC immediately enters into steady state phosphorylation upon the introduction of 5-fold standard KaiA at different time points. The final phosphorylation levels of KaiC are the same for the different perturbation time points. Introducing KaiA at the trough time stimulates the largest transient fluctuation (the maximum phosphorylation level), suggesting that KaiA reaches its maximum activity at this time point. Therefore, the apparent activation-inactivation of KaiA does exist, as proposed by Rust et al. Our relatively complex model may show an explicit mechanism of KaiA activity variation. In effect, the reduction of KaiA activity is mainly attributed to the competition with KaiB (on KaiC binding) and inhibition (or trapping) within KaiABC complex. And all the processes are controlled by KaiC phosphorylation levels, especially those of S431.

### Phase response curves by stimuli of transient changes in KaiA concentration

To make our model more predictive, we further explore the phase response curve (PRC) [45] of the *in vitro* Kai oscillator, using the transient KaiA concentration variation as the stimulus pulse. Specifically, 4-hour pulses are applied to various time points in the circadian cycle as Kai oscillator is free running. At the beginning of the pulse, KaiA concentration changes to a non-standard amount, lasting for four hours, and returns to its standard quantity at the end of the pulse. In this work, four concentration values of KaiA pulses are used: 1/3×, 2/3×, 1.5× and 3× standard amount of KaiA.

**Figure 9:**
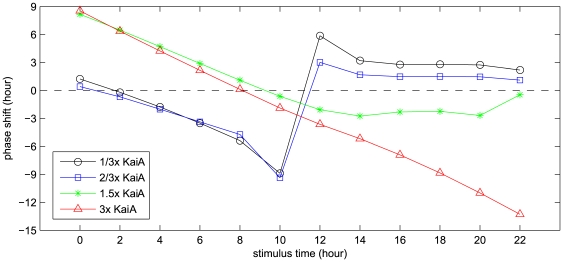
Phase response curves obtained by transient variations in KaiA concentration. Nonstandard KaiA concentrations are used as the 4-hour stimulus pulses, i.e. 1/3×, 2/3×, 1.5× and 3× standard amount of KaiA. Parameter set used is the new one obtained in section 2.6 in Text S1.

The phase shifts under these pulses are shown in Fig. 9 as the function of circadian time, and the raw dynamic phase shift profiles can be found in section 2.10 in Text S1. The zero circadian time, as we defined previously, is the trough time of total KaiC phosphorylation. Under the low-KaiA stimuli (1/3× and 2/3×), the phases of KaiC dynamics delay during most of the phosphorylation course (0 h∼10 h), while the phases advance throughout dephosphorylation. In both 1/3× and 2/3× KaiA pulses, the delayed phase shifts change rapidly and reach the maximum (−10 h) at the circadian time 10 h, whereas the advanced shifts stay around 2 h during most of the dephosphorylation phase. There is a discontinuous transition from delays to advances (at 10 h∼12 h) for 1/3× and 2/3× KaiA stimuli, respectively, which mainly attributes to the choice of zero circadian time. Since the *in vitro* Kai oscillator has no cue of real day and night, the zero circadian time could be chosen arbitrarily.

On the contrary to the 1/3× and 2/3× pulses, the high-KaiA stimuli (1.5× and 3×) cause phase advances mostly throughout phosphorylation and delays during dephosphorylation. The two PRCs by high-KaiA stimuli (1.5× and 3×) are similar during the phosphorylation phase (0∼10 h), while they become quite different as the stimuli are applied in the dephosphorylation phase (10∼22 h). During most of the time in dephosphorylation phase, there are only some slight shifts in the delayed phases by 1.5× KaiA pulses, whereas the 3× KaiA pulses cause continuous increase in phase delays. It suggests that the sensitivity of the phase delay under the high-KaiA pulses depends on the very dose of KaiA applied.

Light, in most cases, is the primary Zeitgeber for the entrainment of circadian rhythms, especially for cyanobacteria, a group of photoautotrophic prokaryotes. Three proteins, CikA, LdpA and Pex are identified to be responsible for the external light-dark stimuli [1]. *In vivo*, they coordinately regulate the transcription of *kaiA* gene to facilitate the day-night entrainment. Therefore, one could assume that the transient change in KaiA concentration *in vitro* may, to some extent, mimic the light or dark pulse applied to the living cell. For instance, increase in KaiA amount could correspond to a light stimulus, while decrease in KaiA concentration mimics a dark pulse. In our results, the dark pulse mimicking PRCs (1/3× and 2/3× KaiA) may differ considerably from those obtained by the real dark pulse *in vivo*. In fact, the condition of free running oscillator *in vivo* is continuous light (LL) in which the concentrations of the Kai proteins fluctuate, especially for KaiC and KaiB, whereas the concentrations of Kai proteins stay mostly unchanged in the *in vitro* Kai pacemaker. Nevertheless, the *in vitro* Kai oscillator could be analogous to the free running *in vivo* pacemaker in continuous dark (DD). Thus, the 1.5× and 3× KaiA-pulse PRCs may accord with those by light-pulse stimuli *in vivo*. We hope the quantitative analysis of PRCs could be helpful for probing the working and entrainment mechanism of the Kai oscillator both i*n vitro* and *in vivo*.

## Discussion

In this work, we propose a multi-layer reaction network to mimic the circadian phosphorylation of KaiC. There are two major features in our model. First, we suggest that one KaiC hexamer can be viewed as a single oscillator. In essence, one hexamer is actually a mesoscopic dissipative subsystem which can exhibit phosphorylation oscillation (even if noisy) in the presence of KaiA and KaiB. Macroscopically, a population of KaiC hexamers constitutes an oscillatory dissipative system which is much more robust than the single KaiC oscillator. Therefore, we can find the self-organization process in a single KaiC oscillator as well as in a bulk phase containing a number of single oscillators. This is probably the most important reason why the rhythm of Kai system is highly robust, especially against fluctuations in temperature and protein concentrations.

Second, we found a dynamic diversity of temporal patterns in KaiC phosphorylation network. The oscillation patterns of the hexameric KaiC phosphoforms in the network exhibit a variety of phases, waveforms and amplitudes. Thus, we deduce that the combination of these diverse phases may produce a kaleidoscopic mode which is helpful to explain the circadian genome-wide gene expression in cyanobacteria. According to the kaleidoscopic mode, combinations of different KaiC phosphoforms in various proportions can produce numerous diverse-phased phosphorylation signals. Meanwhile, due to different random fluctuations in the absolute molecule numbers of KaiC phosphoforms, regulation of these hexamers in gene expressions can swing among deterministic, pseudo-stochastic or stochastic modes, which may be significant to the stochastic gene expressions in the out-of-steady-state system [40]. Additionally, we suppose that 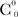 can be considered as a *primary*-*master* oscillator (strictly as an oscillatory output). The other KaiC phosphoforms are probably the *secondary-master* oscillators. 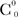 is the major component of KaiC phosphorylation cycle, yet it can not meet the need of the regulation of basal metabolism because it contains null phosphorylation information. The maintenance of physiological functions thus requires the participation of the other KaiC phosphoforms. If so, we deduce that *S. elongates* master phase should approximately be anti-phase with 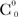. Based on the stochastic kaleidoscopic regulation, it is postulated that Kai oscillator may control the global circadian transcription in coupling with some known mechanisms such as topological changes in chromosome [46],[47], binding to forked DNA [6], interacting with SasA-RpaA and LabA [48],[49] or signaling with sigma factors [1].

Based on our estimation of free energy of activation, temperature compensation can be qualitatively explained as an inherent structural property of a well-coordinated KaiC hexamer (simulation results can be found in section 2.4 in Text S1). Further analysis of the coupling of the three hierarchical energy modes, i.e. local, quasi-local and global, reveals subtle relationships between temperature compensation and the kinetic cooperativity. In wide-type KaiC, the local mode energy may be the coarse-tune component of temperature compensation in circadian KaiC phosphorylation, while the global mode is probably to fine-tune the kinetic cooperativity. The quasi-local mode most likely fine-tunes the temperature compensation and regulates the kinetic cooperativity as the coarse-tune component. Accordingly, we may predict several extreme dynamic behaviors in mutant KaiC phosphorylation. Elimination of global mode (or significant change in quasi-local mode) will result in the loss of kinetic cooperativity, and finally abolishes the oscillation of KaiC phosphorylation, yet the robustness of temperature compensation (considering the steady-state reaction rates) may be still maintained in this case. If a moderate change in interaction energy in local mode occurs, the temperature compensation is no longer robust but an extreme low amplitude, long period and probably noisy oscillation of KaiC phosphorylation may be observable or even the oscillation of KaiC phosphorylation is totally abolished.

The deterministic dynamic simulations in a reduced model confirm that the monomer-shuffling favors the synchronization of KaiC phosphorylation level, but it results in longer period and lower amplitude (refer to section 2.9 in 1). Without introducing an explicit monomer-shuffling process, simulations in the full model obtain very consistent results with a recent experimental study on phase synchronization [43]. We suggest that the phosphorylation level at S431 is mainly responsible for the phase coherence for a population of KaiC hexamers. Due to the limited information about the monomer-shuffling mechanism, it definitely hinders the detailed analyses of this process. Verification of the predictions proposed in the present work and comprehensive elucidation of the KaiC circadian phosphorylation *in vitro* and *in vivo* absolutely depend on the new findings achieved experimentally and theoretically.

## Supporting Information

Text S1 This text contains detailed methods for the full Kai network and additional results.
(2.00 MB PDF)

Table S1 Expressions and descriptions for reaction flows.
(0.02 MB PDF)

Table S2 Relationship between ST and subunit representations.
(0.08 MB PDF)

Table S3 Combinational factors of the triplets in KaiC hexamer.
(0.05 MB PDF)

Table S4 Dissociation constants and rate constants in Kai reaction network.
(0.20 MB PDF)

Table S5 Parameters in the estimation of free energies of all reaction and interaction processes in Kai network.
(0.12 MB PDF)

Video S1 Dynamic node-to-node mass evolution in one complete KaiC phosphorylation cycle. The time for the trough of *Y* is set to be the zero circadian time. Mass evolution (contour map) in LC (left panel), LBC (right panel) and combined (middle panel) layers are shown. The color bar shown on right corresponds to the fraction of the amount of total KaiC hexamer.
(1.16 MB WMV)
