# Supplementary material for: Circadian KaiC Phosphorylation: A Multi-Layer Network
Source: PLoS Comput Biol. 2009 Nov 20;5(11):e1000568. doi: 10.1371/journal.pcbi.1000568 (PMC2773046; doi:10.1371/journal.pcbi.1000568)
Supplement: Text S1 — This text contains detailed methods for the full Kai network and additional results. (2.00 MB PDF) [file pcbi.1000568.s001.pdf]

# Supporting Information for

## Circadian KaiC Phosphorylation: A Multi-layer Network

Congxin Li, Xiaofang Chen, Pengye Wang, Weichi Wang*

*To whom correspondence should be addressed. E-mail: weichi@aphy.iphy.ac.cn

This supporting information contains:

1. Detailed methods for the full network

2. Additional results

3. References

# Table of contents

1. Detailed methods for the full network…………………………………………………….......... 3

1.1 Kinetic equations for the full KaiC reaction network…………………………………………… 3

1.2 Estimation of free energy of activation for phosphorylation

(or dephosphorylation) and Kai protein interactions……………………………………………..… 6

1.2.1 Free energy of activation for KaiC phosphorylation and dephosphorylation………………………………………… 7

1.2.2 Transformation between *ST representation* and *subunit representation*…………………………………….…. 12

1.2.3 Free energy for Kai protein interaction…………………………………………………………...…………. 15

1.2.4 A simple coarse-grained normal mode analysis……………………………………………………..…………. 15

1.2.5 Estimation of parameters……………………………………………………………………………………..23

2. Additional results……………………………………………………………………….. 29

2.1 Functional differentiation between S431 and T432……………………………………………. 29

2.2 Stochastic simulation of the Kai reaction network…………………………….…………………. 30

2.3 Diverse circadian patterns of KaiC phosphoforms……………………………………….……. 31

2.4 Temperature compensation in Kai system……………………………………………….……….. 32

2.5 Bifurcation diagrams for variations in concentration of KaiA and KaiB……………………..…….. 33

2.6 Dynamics of Kai oscillator under concerted changes in protein concentrations………………………..34

2.7 Synchronization of different phased Kai samples………………………………..………………. 36

2.8 Simulation of mixing Kai samples with non-standard concentrations……………………………....37

2.9 Reduced model and monomer shuffling……………………………………………….……………. 39

2.10 Dynamic phase shifts by transient variations in KaiA concentration………………………………….40

3. References…………………………………………………………………….…………. 43

# 1. Detailed methods for the full network

This section contains two parts. The first part (1.1) illustrates the kinetic equations for the full Kai network. The second part (1.2) introduces the quantitative method for the estimation of all the kinetic constants.

## 1.1 Kinetic equations for the full KaiC reaction network

To describe the dynamics of the Kai system, we use the *ST representation* $C_s^t$ to establish the full network and the corresponding kinetic equations. As defined in the main text, $C_s^t$ stands for one KaiC hexamer with *s* S431 and *t* T432 site(s) phosphorylated. *ST representation* is convenient to outline a clear topological structure of KaiC network shown in Fig. S1A-B.

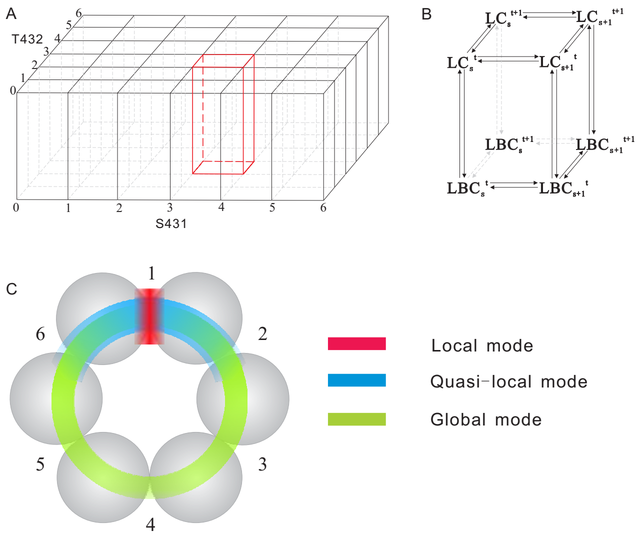


**Fig. S1.** Main schemes for Kai full network. (A) The framework of the two-layer network. The upper network represents the LC layer, while the bottom the LBC layer. (B) Detailed description of an element unit (in red) in Fig. S1A. (C) Illustration for the hierarchical decomposition of the free energy of activation. The local mode (red) is mainly restricted at the reaction interface. The quasi-local mode (blue) can be dispersed within the two co-interface subunits even up to the border of next interface. The global mode (green) propagates throughout the whole KaiC hexamer.

Deterministic mass action law and rapid equilibrium method are applied to the full reaction network. KaiA-KaiC binding is described by rapid equilibrium method and we have:

$$[\mathrm{C}_s^t] = \frac{Ka_s^t}{Ka_s^t + [\mathrm{A}]}[\mathrm{LC}_s^t] \tag{6}$$

$$[\mathrm{AC}_s^t] = \frac{[\mathrm{A}]}{Ka_s^t + [\mathrm{A}]}[\mathrm{LC}_s^t] \tag{7}$$

where $[\mathrm{LC}_s^t] = [\mathrm{C}_s^t] + [\mathrm{AC}_s^t]$, and $Ka_s^t$ is the dissociation constant.

The reaction rate of association-dissociation of KaiC and KaiB (two KaiB dimers binding to one KaiC hexamer) is described by:

$$JBC_s^t = kb_s^t[\mathrm{C}_s^t][\mathrm{B}]^2 - kdb_s^t[\mathrm{B}_2\mathrm{C}_s^t] \tag{8}$$

where $kb_s^t$ and $kdb_s^t$ are the rate constants for KaiB-KaiC association and dissociation, respectively. The formation of KaiABC is depicted by rapid binding of KaiA to KaiBC, and three KaiA dimers binds to KaiBC in a sequential manner. Moreover, we assume the affinity of KaiA to KaiBC is not influenced by the number of KaiA already bound on KaiBC. Using rapid equilibrium method, one can have:

$$[\mathrm{B}_2\mathrm{C}_s^t] = \frac{(Kba_s^t)^3}{(Kba_s^t)^3 + (Kba_s^t)^2[\mathrm{A}] + (Kba_s^t)[\mathrm{A}]^2 + [\mathrm{A}]^3}[\mathrm{LBC}_s^t] \tag{9}$$

$$[\mathrm{AB}_2\mathrm{C}_s^t] = \frac{(Kba_s^t)^2[\mathrm{A}]}{(Kba_s^t)^3 + (Kba_s^t)^2[\mathrm{A}] + (Kba_s^t)[\mathrm{A}]^2 + [\mathrm{A}]^3}[\mathrm{LBC}_s^t] \tag{10}$$

$$[\mathrm{A}_2\mathrm{B}_2\mathrm{C}_s^t] = \frac{(Kba_s^t)[\mathrm{A}]^2}{(Kba_s^t)^3 + (Kba_s^t)^2[\mathrm{A}] + (Kba_s^t)[\mathrm{A}]^2 + [\mathrm{A}]^3}[\mathrm{LBC}_s^t] \tag{11}$$

$$[\mathrm{A}_3\mathrm{B}_2\mathrm{C}_s^t] = \frac{[\mathrm{A}]^3}{(Kba_s^t)^3 + (Kba_s^t)^2[\mathrm{A}] + (Kba_s^t)[\mathrm{A}]^2 + [\mathrm{A}]^3}[\mathrm{LBC}_s^t] \tag{12}$$

Where $[\mathrm{LBC}_s^t] = [\mathrm{B}_2\mathrm{C}_s^t] + [\mathrm{AB}_2\mathrm{C}_s^t] + [\mathrm{A}_2\mathrm{B}_2\mathrm{C}_s^t] + [\mathrm{A}_3\mathrm{B}_2\mathrm{C}_s^t]$, and $Kba_s^t$ is the dissociation

constant for each reaction step between KaiA and KaiBC. The association and dissociation of KaiB-KaiC are much slower than those of KaiA-KaiC. Additionally, the amount of KaiAC is less than that of KaiBC [1]. Thus, for simplicity, the other pathway of KaiABC formation, namely $\mathrm{AC} + \mathrm{B} \leftrightarrow \mathrm{ABC}$, is not included.

Due to the rapid equilibrium between KaiC and KaiAC, their phosphorylation and dephosphorylation processes can be technically described by the dynamics of the combined mode $\mathrm{LC}_s^t$ ($\mathrm{LC}$ for short). The same technique is also used in $\mathrm{LBC}_s^t$ ($\mathrm{LBC}$ for short) layer. We neglect several reaction processes because of their relative small rates (comparing to their reaction rates in the opposite direction), i.e. phosphorylation (at both S431 and T432) of free KaiC and KaiBC, dephosphorylation (at both S431 and T432) of KaiAC.

The kinetic equations for the whole reaction network are as follows:

$$\frac{d[\mathrm{LC}_s^t]}{d\tau} = \eta_{t0}(Japt_s^{t-1} - Jdpt_s^t) + \eta_{t6}(Jdpt_s^{t+1} - Japt_s^t) + \eta_{s0}(Japs_{s-1}^t - Jdps_s^t) + \eta_{s6}(Jdps_{s+1}^t - Japs_s^t) - JBC_s^t \tag{13}$$

$$\begin{aligned}\frac{d[\mathrm{LBC}_s^t]}{d\tau} &= \eta_{t0}(Jabpt_s^{t-1} - Jbdpt_s^t - Jabdpt_s^t) + \eta_{t6}(Jbdpt_s^{t+1} + Jabdpt_s^{t+1} - Jabpt_s^t) \\ &\quad + \eta_{s0}(Jabps_{s-1}^t - Jbdps_s^t - Jabdps_s^t) + \eta_{s6}(Jbdps_{s+1}^t + Jabdps_{s+1}^t - Jabps_s^t) + JBC_s^t\end{aligned} \tag{14}$$

$$\eta_{kl} = \begin{cases} 1 & k \neq l \\ 0 & k = l \end{cases}$$

$$[\mathrm{B}]_\mathrm{T} = [\mathrm{B}] + 2\sum_{s,t}[\mathrm{LBC}_s^t] \tag{15}$$

$$[\mathrm{A}]_\mathrm{T} = [\mathrm{A}] + \sum_{s,t}([\mathrm{AC}_s^t] + [\mathrm{AB_2C}_s^t] + 2[\mathrm{A_2B_2C}_s^t] + 3[\mathrm{A_3B_2C}_s^t]) \tag{16}$$

where $s,t = 0,...,6$

There are totally 100 equations for the full Kai network. 98 (2×49) of them are

ordinary differential equations for phosphorylation and dephosphorylation reactions. As $s$ and $t$ goes from 0 to 6, respectively, two sets of ordinary differential equations can be automatically generated from Eq. 13 and Eq. 14. Each set contains 49 equations, describing the dynamics of 49 nodes in LC or LBC layer. The notation $\eta_{kl}$ is useful to generate the correct forms of equations describing the nodes on the edges of the network. Other two algebraic equations (Eq. 15 and 16) represent the mass conservation law for KaiB and KaiA in the whole network, respectively. The mass conservation for KaiC can be examined by summing the first two sets of ODEs. The expressions and descriptions of reaction flows $J$ in the equations are listed in Table S1. Note that $AB_2C_s^t$, $A_2B_2C_s^t$ and $A_3B_2C_s^t$, share the same rate constants of phosphorylation and dephosphorylation.

## 1.2 Estimation of free energy of activation for phosphorylation (or dephosphorylation) and Kai protein interactions

The total number of kinetic rate constants and dissociation constants is 616 for the full Kai network. However, it is unreasonable to manually assign the values to 616 parameters. Thus, we introduce a quantitative method to correlate the kinetic parameters with KaiC phosphorylation states.

Briefly, we use an approximate method to literally calculate all the reaction rates and dissociation constants in our model. It is difficult for us to directly compute the values of kinetic constants for every hexameric KaiC phosphoform ab initio. However, we can deduce the intrinsic correlations among KaiC phosphorylation (or

dephosphorylation) rates and among the dissociation constants of Kai protein interactions. In principle, the network represents the time evolution of one KaiC hexamer from $C_0^0$ to $C_6^6$, so the kinetic property of each state of KaiC (each node in the network) is inherently correlated with others. This characteristic of Kai network is the basis of our estimation. Technically, we analyze the relations between free energies of activation and KaiC phosphorylation levels based on intra- and inter-subunit interactions within one KaiC hexamer. Then, we estimate the quantity scale of the free energies so that they fall into the scale of free energies of general biochemical reactions, e.g. elementary Ser/Thr phosphorylation, ATP hydrolysis. The obvious strength of our method is that it can automatically generate 616 kinetic constants (see Table S4) with only 88 basic parameters ($\lambda$, $\sigma$ and $\omega$, see Table S5) in our model. In fact, the 88 parameters are not fully independent and must be chosen under the strict reasoning and extra constraints. The qualitative and semi-quantitative analyses of constraints among the 88 parameters are described in section 1.2.5.

Further, we are aware that the high dimensional variable and parameter spaces in our work may hinder the practical use of the model in further experimental and theoretical studies. In our pending work, based on the works by Winfree [2] and Kuramoto [3], we could get a simplified yet useful model which can be integrated into the future *in vivo* circadian model in cyanobacteria.

### 1.2.1 Free energy of activation for KaiC phosphorylation and dephosphorylation

The finding of ATPase activity on KaiC hexamer encourages us to hypothesize the

ATP hydrolysis by KaiC ATPase is the main driving force to regulate (not initiate) the circadian KaiC phosphorylation. As shown in Fig. 1B (in the main text), an isolated phosphorylation site has a high free energy of activation, and it is difficult to self-regulate its own phosphorylation (or dephosphorylation) because there is no interactions with its neighbors. Taking the advantage of the ATP hydrolysis, isolated KaiC monomers can form stable and coordinated hexamer at both ground state and transition state. Therefore, the apparent free energy of activation for a KaiC hexamer contains, in principle, all the structural information of the six subunits, which may serve as the basis for self-regulated phosphorylation (or dephosphorylation).

The decomposition of free energy may provide us a deep insight into the hierarchical energy landscape in KaiC circadian phosphorylation. In general, no adequate evidence is available to show what type(s) of thermodynamic change(s) (entropic and/or enthalpic) can be defined corresponding to the energy diffusion modes. High frequency modes are generally localized in proteins and thus hinder the intra- and/or inter-molecular communications [4]. However, the model based on the experiment of protein-DNA interactions suggests that a coupling of fast, local modes with the slow, global modes in favor of a potential amplification of entropic allostery, and such coupling gives rise to large compensation in enthalpy and entropy in proteins [5]. We infer that the wide-type KaiC of *S. elongatus* PCC 7942 accords with the last type in energy distribution at transition state. Here, we may deal with the KaiC hexamer as an energy carrier, and an information processor.

As illustrated in Fig. S1C, We roughly dissect the free energy change into three

hierarchical levels: the largest portion localized at the reaction interface (local mode), the relative small part of the energy that can be dispersed in the two co-interface subunits even up to the border of next interface (quasi-local mode), and the smallest portion that propagates throughout the whole KaiC hexamer (global mode). This three-leveled decomposition of free energy is definitely not unique, and a later analytic derivation in section 1.2.4 suggests the existence of much fine levels of interaction energies.

Based on the assumptions above, we can estimate the form of $\Delta U_\chi$ and $\Delta U_\tau$ in Eq. 1 (in the main text). For clarity, interfaces in one KaiC hexamer are numbered from 1 to 6 clockwise, assigned as $\mathrm{I}_k$ $(k{=}1 \text{ to } 6)$, and $\mathrm{I}_1$ is assumed to be the reaction (phosphorylation or dephosphorylation) interface, shown in Fig. S1C. $\Delta U_\chi$ and $\Delta U_\tau$ can be expressed as:

$$\Delta U_\chi = \sum_{i=1}^{6} \Delta E_i + H_\chi \tag{17}$$

$$\Delta U_\tau = \Delta E_1^{\neq} + \sum_{i=2}^{6} \Delta E_i + H_\tau \tag{18}$$

where $\Delta E_i$ is the local mode energy of each non-reaction interface that mainly serves as the hinge holding the subunits together to form a stabilized and integrated hexamer, and $\Delta E_1^{\neq}$ plays the same part for the transition state interface (here for $\mathrm{I}_1$); $H_\chi$ ($H_\tau$) is the sum of the triple-interface-coupled quasi-local mode energy and global mode energy for ground state (transition state). Using Eqs.17-18, we can rewrite Eq.1 as

$$\Delta G^{\neq} = \Delta G^{\neq *} + \Delta\Delta E_1^{\neq} + (H_\tau - H_\chi) \tag{19}$$

where $\Delta\Delta E_1^{\neq} = \Delta E_1^{\neq} - \Delta E_1$

Assuming all the multi-body ("body" here refers to "interface") interactions are harmonic, $H_\chi$ has the form

$$H_\chi = H_0(\lambda_6\lambda_1\lambda_2 + \lambda_1\lambda_2\lambda_3 + \lambda_2\lambda_3\lambda_4 + \lambda_3\lambda_4\lambda_5 + \lambda_4\lambda_5\lambda_6 + \lambda_5\lambda_6\lambda_1 + \omega\lambda_1\lambda_2\lambda_3\lambda_4\lambda_5\lambda_6) \quad (20)$$

The product $\lambda_i\lambda_j\lambda_k$ refers to the effect of interaction among three adjacent interfaces, where $\lambda_i$ is the harmonic factor (dimensionless) weighing the contribution of each interface to multi-interface interactions. The last term in the bracket in Eq. 20 represents the global mode (related to all the six interfaces) interaction, where $\omega$ is the transmission factor defining the relative quantity scale of the global mode energy. $H_0$ characterizes the basic harmonic energy scale.

Similarly, one can have

$$H_\tau = H_0[\eta(6,1^{\neq},2)\lambda_6\lambda_1^{\neq}\lambda_2 + \eta(1^{\neq},2,3)\lambda_1^{\neq}\lambda_2\lambda_3 + \lambda_2\lambda_3\lambda_4 + \lambda_3\lambda_4\lambda_5 + \lambda_4\lambda_5\lambda_6 + \eta(5,6,1^{\neq})\lambda_5\lambda_6\lambda_1^{\neq} + \omega\lambda_1^{\neq}\lambda_2\lambda_3\lambda_4\lambda_5\lambda_6] \quad (21)$$

$\lambda_i$ is the same as that in $H_\chi$, whereas $\lambda_1^{\neq}$ stands for harmonic factor for the interface at transition state. Particularly, in contrast to ground state, the transition state of KaiC hexamer exhibits an asymmetry energy distribution due to the sole transition state interface. It directly influences the $I_1$-involved triplet (3-interface) interaction energy. For simplicity, only when $I_1$ is located at the center in the triplet can it bring about considerable change in the energy at the transition state, whereas the contributions of triplets with $I_1$ on the side are almost equal to those of the triplets in the ground state. $\eta(i,j,k)$ is used to demonstrate this effect: $\eta(i,j^{\neq},k)=1$, $\eta(i^{\neq},j,k) \approx \lambda_j / \lambda_j^{\neq}$ and $\eta(i,j,k^{\neq}) \approx \lambda_k / \lambda_k^{\neq}$. Hence,

$$H_\tau - H_\chi = H_0(\lambda_1^{\neq} - \lambda_1)\lambda_6\lambda_2 + \omega H_0(\lambda_1^{\neq} - \lambda_1)\lambda_2\lambda_3\lambda_4\lambda_5\lambda_6 \qquad (22)$$

Substituting Eq. 22 into Eq. 19, the final free energy of activation can be derived:

$$\Delta G^{\neq} = \Delta G^{\neq *} + \Delta\Delta E_1^{\neq}(1 + \sigma_1^{\neq}\lambda_6\lambda_2 + \omega\sigma_1^{\neq}\lambda_2\lambda_3\lambda_4\lambda_5\lambda_6) \qquad (23)$$

where $\sigma_1^{\neq} = H_0(\lambda_1^{\neq} - \lambda_1)/\Delta\Delta E_1^{\neq}$. Because $\sigma_1^{\neq}$ is linearly related to $\lambda_1^{\neq}$, we set $\sigma_1^{\neq}$ to be a fundamental parameter instead of $\lambda_1^{\neq}$, and reassign the name of $\sigma_1^{\neq}$ as the harmonic factor at transition state.

In the *subunit representation*, Eq. 23 can be rewritten in a more generalized form, for example, the free energy of activation of $m_{00}$ phosphorylation at T432 is shown by:

$$\Delta G_{00\mathrm{Tp}}^{\neq} = \Delta G_{\mathrm{p}}^{\neq *} + \Delta\Delta E_{\mathrm{p}}^{\neq}[1 + \sigma_{00\mathrm{Tp}}^{\neq}\lambda_{00}^{i}\lambda_{01}^{j}\lambda_{10}^{k}\lambda_{11}^{l} + \omega\sigma_{00\mathrm{Tp}}^{\neq}\lambda_{00}^{\alpha-1}\lambda_{01}^{\beta}\lambda_{10}^{\gamma}\lambda_{11}^{\delta}] \qquad (24)$$

where $\lambda_{00}$, $\lambda_{01}$, $\lambda_{10}$ and $\lambda_{11}$ are the ground state harmonic factors of $m_{00}$, $m_{01}$, $m_{10}$ and $m_{11}$, respectively. $\sigma_{00\mathrm{Tp}}^{\neq}$ is the transition state harmonic factor for $m_{00}$ phosphorylation at T432. $\alpha$, $\beta$, $\gamma$, $\delta$ and $i$, $j$, $k$, $l$ are non-negative integers under the constraints $\alpha \geq 1$, $\alpha + \beta + \gamma + \delta = 6$, $i + j + k + l = 2$, $i < \alpha, j \leq \beta, k \leq \gamma, l \leq \delta$. Additionally, the free energy of activation of $m_{11}$ dephosphorylation at S431 is

$$\Delta G_{11\mathrm{Sdp}}^{\neq} = \Delta G_{\mathrm{d}}^{\neq *} + \Delta\Delta E_{\mathrm{d}}^{\neq}[1 + \sigma_{11\mathrm{Sdp}}^{\neq}\mu_{00}^{i}\mu_{01}^{j}\mu_{10}^{k}\mu_{11}^{l} + \omega\sigma_{11\mathrm{Sdp}}^{\neq}\mu_{00}^{\alpha}\mu_{01}^{\beta}\mu_{10}^{\gamma}\mu_{11}^{\delta-1}] \qquad (25)$$

where $\Delta G_{\mathrm{d}}^{\neq *}$ is the intrinsic free energy of activation for dephosphorylation which keeps constant in the dephosphorylation of KaiC and its complexes. $\Delta\Delta E_{\mathrm{d}}^{\neq}$ is the counterpart of $\Delta\Delta E_{\mathrm{p}}^{\neq}$, and each KaiC component (free KaiC and its complexes) has its own quantity of $\Delta\Delta E_{\mathrm{d}}^{\neq}$. Here, $\mu$ and $\lambda$ satisfy the relationships $\mu_{00}\lambda_{00} = \mu_{01}\lambda_{01} = \mu_{10}\lambda_{10} = \mu_{11}\lambda_{11} = 1$. These relationships, though not a unique form,

suggest that the same interface (or subunit) has opposite effects on phosphorylation and dephosphorylation. Note that in the term of triplet (3-interface) energy, the two $\lambda_{ij}$ (the second term in the bracket in Eq. 24) must correspond to two peripheral interfaces with the transition state interface in the center. Similarly, other forms of the free energy of activation can be obtained as above.

There are totally 88 basic parameters, such as $\lambda$, $\sigma$ and $\omega$ etc (see Table S5). The values of these parameters should not be chosen arbitrarily due to the intrinsic constraints. Further analyses of the parameter estimation can be found later in section 1.2.5.

### 1.2.2 Transformation between *ST representation* and *subunit representation*

The transformation between the two representations is based on three ingredients. First, two subunits in *subunit representation* can undergo one same reaction in *ST representation*, e.g. both $m_{00}$ and $m_{01}$ are able to be phosphorylated at T432. Second, in $C_{\alpha,\beta,\gamma,\delta}$ there are various combinations of triplets including one certain transition interface (refer to Table S3). Third, one $C_s^t$ generally contains multiple $C_{\alpha,\beta,\gamma,\delta}$, and this degeneracy is shown in detail in Table S2.

For a given KaiC hexamer (e.g. $3m_{00}+m_{01}+m_{10}+m_{11}$, where $\alpha$=3, and $\beta$=$\gamma$=$\delta$=1), the triplet can have several combinations, and each combination accompanies a statistical factor ${}^{pq}N_{\alpha,\beta,\gamma,\delta}^{i,j,k,l}$ ($p$, $q$=0,1) whose value depends on $\alpha$, $\beta$, $\gamma$, $\delta$ and $i$, $j$, $k$, $l$. For instance, the combination factor of three-identical-monomer triplet (e.g. $m_{00}$-$\mathbf{m_{00}}$-$m_{00}$, the $m_{00}$ marked bold is the reaction interface) is $\binom{\alpha}{1}\binom{\alpha-1}{2}$, while

that of three different monomers (e.g. $m_{01}$-$\mathbf{m_{11}}$-$m_{10}$) is $\binom{\delta}{1}\binom{\beta}{1}\binom{\gamma}{1}$. Details can be found in Table S3. The statistical factor is independent of the specific reaction and only determined by the combinations of four types of subunits in the triplet, for instance, ${}^{10}N^{i,j,k,l}_{\alpha,\beta,\gamma,\delta}$ is the statistical factor for both $m_{10}$ phosphorylation at S431 and its dephosphorylation at T432. The combination factor $N$ thus establishes a natural statistical correlations between dual-site phosphorylation or/and dephosphorylation.

To obtain the rate constants in the whole KaiC reaction network, the free energies estimated above have to be transformed from *subunit representation* ($C_{\alpha,\beta,\gamma,\delta}$ with 84 elements) into *ST representation* ($C_s^t$ with 49 elements). The transformation can bring about statistical errors because of the degeneracy in which several $C_{\alpha,\beta,\gamma,\delta}$ correspond to one $C_s^t$. The degree of degeneracy, assigned as $W$, ranges from 1 (no degeneracy) to 4, and determined by ($s$, $t$) in $C_s^t$. For instance, $C_0^0$ only has one combinatorial form, where $\alpha = 6, \beta = 0, \gamma = 0, \delta = 0$. Yet for $C_3^3$, there are four possible combinatorial forms, where

$$\alpha_1 = 0, \beta_1 = 3, \gamma_1 = 3, \delta_1 = 0;$$

$$\alpha_2 = 1, \beta_2 = 2, \gamma_2 = 2, \delta_2 = 1;$$

$$\alpha_3 = 2, \beta_3 = 1, \gamma_3 = 1, \delta_3 = 2;$$

$$\alpha_4 = 3, \beta_4 = 0, \gamma_4 = 0, \delta_4 = 3.$$

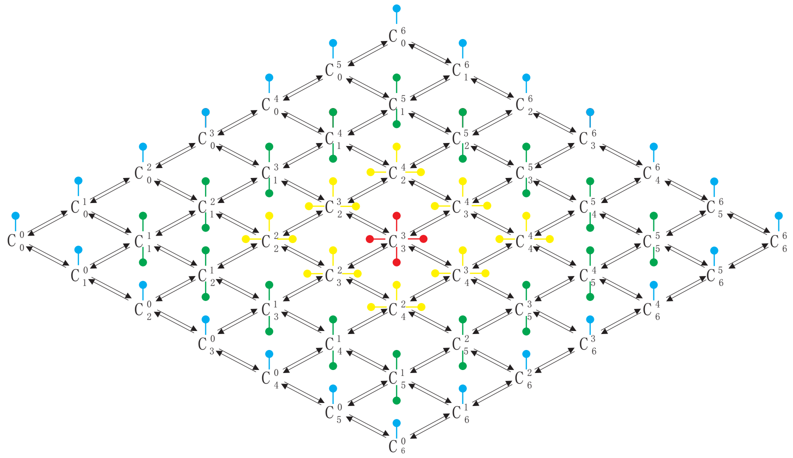

Fig. S2. Illustration for degeneration relationship between *ST representation* and *subunit representation*. Each node on the boundary loop of KaiC network has no degeneration, represented by one balloon. Nodes with degree of degeneration as 2, 3 and 4 are labeled by two, three and four balloons, respectively. The network shows a multi-shell structure according to the degrees of degeneration, shown by different colors.

A general picture is shown in Fig. S2. Numerated the reaction loop in the network from outside to inside, the first loop (the boundary) in the network is not degenerate ($W$=1), $W$=2 in the second loop (such as the pathway along $C_1^1 \rightarrow C_1^6$), $W$=3 in the third loop and $W$=4 only for the central node $C_3^3$. For the nodes on the boundary of the network, there is no statistical error under the transformation between the two representations, whereas $C_3^3$ inevitably causes the largest statistical error. To reduce the errors, a statistical weight $D_{\alpha_m,\beta_m,\gamma_m,\delta_m} \equiv 6!/(\alpha_m!\beta_m!\gamma_m!\delta_m!)$ is introduced using a mean-field method to the distribution of phosphates or subunits in each single KaiC hexamer, It calculates the fractions of the different $C_{\alpha,\beta,\gamma,\delta}$ corresponding to one specific $C_s^t$, where $m$ varies from 1 to $W$ (details in Table S2). Thus, the weighed average can be used to obtain the final free energies in the *ST representation*, shown as below.

$$\Delta G^{\neq}_{\mathrm{Tp}}(s,t)=\frac{\sum_m \sum_{i,j,k,l} D_{\alpha_m,\beta_m,\gamma_m,\delta_m}[({}^{00}N^{i,j,k,l}_{\alpha_m,\beta_m,\gamma_m,\delta_m})({}_{00\mathrm{Tp}}^{\neq}\Delta G^{i,j,k,l}_{\alpha_m,\beta_m,\gamma_m,\delta_m})+({}^{01}N^{i,j,k,l}_{\alpha_m,\beta_m,\gamma_m,\delta_m})({}_{01\mathrm{Tp}}^{\neq}\Delta G^{i,j,k,l}_{\alpha_m,\beta_m,\gamma_m,\delta_m})]}{\sum_m \sum_{i,j,k,l} D_{\alpha_m,\beta_m,\gamma_m,\delta_m}({}^{00}N^{i,j,k,l}_{\alpha_m,\beta_m,\gamma_m,\delta_m}+{}^{01}N^{i,j,k,l}_{\alpha_m,\beta_m,\gamma_m,\delta_m})} \tag{26}$$

$$\Delta G^{\neq}_{\mathrm{Sp}}(s,t)=\frac{\sum_m \sum_{i,j,k,l} D_{\alpha_m,\beta_m,\gamma_m,\delta_m}[({}^{00}N^{i,j,k,l}_{\alpha_m,\beta_m,\gamma_m,\delta_m})({}_{00\mathrm{Sp}}^{\neq}\Delta G^{i,j,k,l}_{\alpha_m,\beta_m,\gamma_m,\delta_m})+({}^{10}N^{i,j,k,l}_{\alpha_m,\beta_m,\gamma_m,\delta_m})({}_{10\mathrm{Sp}}^{\neq}\Delta G^{i,j,k,l}_{\alpha_m,\beta_m,\gamma_m,\delta_m})]}{\sum_m \sum_{i,j,k,l} D_{\alpha_m,\beta_m,\gamma_m,\delta_m}({}^{00}N^{i,j,k,l}_{\alpha_m,\beta_m,\gamma_m,\delta_m}+{}^{10}N^{i,j,k,l}_{\alpha_m,\beta_m,\gamma_m,\delta_m})} \tag{27}$$

$$\Delta G^{\neq}_{\mathrm{Tdp}}(s,t)=\frac{\sum_m \sum_{i,j,k,l} D_{\alpha_m,\beta_m,\gamma_m,\delta_m}[({}^{10}N^{i,j,k,l}_{\alpha_m,\beta_m,\gamma_m,\delta_m})({}_{10\mathrm{Tdp}}^{\neq}\Delta G^{i,j,k,l}_{\alpha_m,\beta_m,\gamma_m,\delta_m})+({}^{11}N^{i,j,k,l}_{\alpha_m,\beta_m,\gamma_m,\delta_m})({}_{11\mathrm{Tdp}}^{\neq}\Delta G^{i,j,k,l}_{\alpha_m,\beta_m,\gamma_m,\delta_m})]}{\sum_m \sum_{i,j,k,l} D_{\alpha_m,\beta_m,\gamma_m,\delta_m}({}^{10}N^{i,j,k,l}_{\alpha_m,\beta_m,\gamma_m,\delta_m}+{}^{11}N^{i,j,k,l}_{\alpha_m,\beta_m,\gamma_m,\delta_m})} \tag{28}$$

$$\Delta G^{\neq}_{\mathrm{Sdp}}(s,t)=\frac{\sum_m \sum_{i,j,k,l} D_{\alpha_m,\beta_m,\gamma_m,\delta_m}[({}^{01}N^{i,j,k,l}_{\alpha_m,\beta_m,\gamma_m,\delta_m})({}_{01\mathrm{Sdp}}^{\neq}\Delta G^{i,j,k,l}_{\alpha_m,\beta_m,\gamma_m,\delta_m})+({}^{11}N^{i,j,k,l}_{\alpha_m,\beta_m,\gamma_m,\delta_m})({}_{11\mathrm{Sdp}}^{\neq}\Delta G^{i,j,k,l}_{\alpha_m,\beta_m,\gamma_m,\delta_m})]}{\sum_m \sum_{i,j,k,l} D_{\alpha_m,\beta_m,\gamma_m,\delta_m}({}^{01}N^{i,j,k,l}_{\alpha_m,\beta_m,\gamma_m,\delta_m}+{}^{11}N^{i,j,k,l}_{\alpha_m,\beta_m,\gamma_m,\delta_m})} \tag{29}$$

### 1.2.3 Free energy for Kai protein interaction

For simplicity, the spatial arrangements of KaiA, KaiB bound to KaiC hexamer are not considered. We assume the free energy differences between free KaiC and Kai complexes are related to the state of individual subunit and the global mode interactions, accordingly one has:

$$\Delta G_{\alpha,\beta,\gamma,\delta}=\alpha\Delta G_{00}+\beta\Delta G_{01}+\gamma\Delta G_{10}+\delta\Delta G_{11}+\Psi h_{00}^{\alpha}h_{01}^{\beta}h_{10}^{\gamma}h_{11}^{\delta} \tag{30}$$

where $\Delta G_{00}$, $\Delta G_{01}$, $\Delta G_{10}$ and $\Delta G_{11}$ represent contributions of four types of subunits, and the last term in Eq. 30 describes a global mode interaction for Kai protein binding, in which $h_{ij}$ ($i,j$ = 0,1) is the harmonic factor and $\Psi$ the basic global mode energy.

The corresponding binding energy in *ST representation* can be obtained by:

$$\Delta G^{\mathrm{ST}} = \frac{\sum_m D_{\alpha_m,\beta_m,\gamma_m,\delta_m} \Delta G_{\alpha_m,\beta_m,\gamma_m,\delta_m}}{\sum_m D_{\alpha_m,\beta_m,\gamma_m,\delta_m}} \tag{31}$$

where $\Delta G_{\alpha_m,\beta_m,\gamma_m,\delta_m}$ is expressed in Eq. 30. The dissociation constant is thus obtained by $K = K_0 e^{-\Delta G^{\mathrm{ST}}/k_{\mathrm{B}}T}$. In this work, KaiB-KaiC association-dissociation must obey the mass action law. We assign the rate constants of forward association as: $kb = kb_0 e^{\Delta G^{\mathrm{ST}}/k_B T}$, and let dissociation rate constants of KaiB-KaiC share the same value $kdb = kdb_0$. Parameters in the free energy estimation are listed in Table S5.

### 1.2.4 A simple coarse-grained normal mode analysis

In this section, a simplified coarse-grained normal mode analysis is performed to support the hierarchical decomposition of free energy adopted in our model. This is a relative independent part of the free energy estimation. The derivations in this section are not used in the practical calculation of rate constants in our work, because they are so complicated and may involve more parameters. These derivations can be conditionally approximated to our equations for free energy mentioned above. The basic procedures are all based on the works of Hawkins and McLeish [5,6].

Normal mode analysis (NMA) is one of the powerful computation methods in exploring protein-protein interaction [7,8]. NMA decomposes complex motion into a sum of independent vibration modes, and uses the harmonic approximation to the potential *U*. Based on the assumption that the displacements of the atoms about their equilibrium positions are small, the harmonic approximation of Newton's equation of

motion can be written as:

$$\mathbf{M}\ddot{\mathbf{x}} = -\mathbf{K}\mathbf{x} \tag{32}$$

where $\mathbf{x}$ is the vector of the 3$N$ particles' displacements, $N$ is the particle number. $\mathbf{M}$ is the inertial matrix, $\mathbf{K}$ is the matrix defined by $K_{ij} = \frac{\partial^2 U(x_1,...,x_{3N})}{\partial x_i \partial x_j}$. The Hamiltonian of the system is given by

$$H = \frac{1}{2}\mathbf{p}^{\mathbf{T}}\mathbf{M}^{\mathbf{-1}}\mathbf{p} + \frac{1}{2}\mathbf{x}^{\mathbf{T}}\mathbf{K}\mathbf{x} \tag{33}$$

where $\mathbf{p}$ stands for the momentum vector.

The partition function is then

$$Z = \int_{-\infty}^{+\infty} e^{-H/(k_{\mathrm{B}}T)}\mathbf{dxdp} = (2\pi k_B T)^d (|\mathbf{M}^{-1}||\mathbf{K}|)^{-1/2} \tag{34}$$

where $d$ is the number of degrees of freedom in the model.

Assuming isothermal changes and any change in the protein mass is negligible, the free energy is then

$$G = -k_{\mathrm{B}}T\ln Z = \frac{1}{2}k_{\mathrm{B}}T\ln|\mathbf{K}| + \text{constant} \tag{35}$$

Thus, $\mathbf{K}$ is crucial to the analysis of the free energy in protein-protein interaction. Eqs. 32-35 are derived by Hawkins and McLeish [5,6].

In KaiC hexamer, the C terminal domain is responsible for phosphorylation, and it has a flexible conformation that may facilitate the intra- and inter-subunit interactions. The N terminal of KaiC has a more rigid structure that hardly tends to directly regulate the phosphorylation in the C terminal [9]. The significance of N terminal is to maintain the integrity and stability of the KaiC hexamer by interacting with ATP. For simplicity, we only consider the C terminal domain in the normal mode analysis for KaiC phosphorylation. A full model that contains both N and C terminal domains

would certainly give more insights into KaiC dynamics. For example, simulations in monomer shuffling definitely require the interplay between N and C terminal domains. Currently, we have difficulties in dealing with this large and complex KaiC system, so here we only study the simplified model containing only C terminal domain. A dual domain model is our pending work.

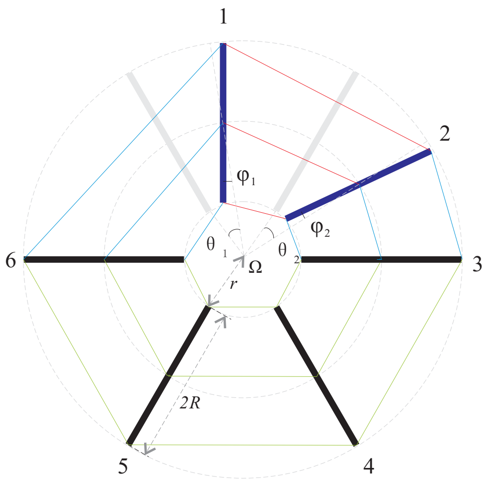


**Fig. S3.** Illustration of 2D coarse-grained model of KaiC hexamer. The solid rods represent the six KaiC subunits. Two adjacent rods are connected by three springs at the center and two tips. The motion of the center of each rod is constrained in a circle orbit, and each rod can also spin about its own center. For instance, No.1 rod (deep blue) moves off its equilibrium position (solid grey rod) by rotating $\theta_1$ around $\Omega$ (the core of the system) and spinning $\varphi_1$ with respect to the center of No.1 rod. Here, we assign that interface of rod 1 and 2 is in the transition state. The changes in spring constants are different among the six interfaces, indicated by their colors.

A 2D coarse-grained model is built for normal mode analysis, as shown in Fig. S3. The rigid rods, each with the length of $2R$, represent the six KaiC subunit (C terminal), and every two adjacent rods are connected with three different springs (using thin solid lines for simplicity). One spring links the centers of the two rods. The other two springs respectively tie the inner and outer ends of the two neighboring rods. Three concentric circles are helpful in the analysis. The radius of the inner circle is $r$, the

distance from the core of the system Ω to the nearest end of the rod. The movement of the center of the rod is constrained in the middle circle, and the degree of freedom of each rod is two, rotation around Ω and spin about its own center. Two independent variables are used to describe each rod (12 overall): $\theta_i$ the angle of the rod's center move away from its original position (in the resting state), and $\varphi_i$ the spin angle, $i$ ranging from 1 to 6; and we define the positive rotation direction is clockwise.

We assign that phosphorylation (or dephosphorylation) taking place at the interface between rod 1 and rod 2 (red springs), the displacement of the middle spring is,

$$\Delta L_{1,2}^{\mathrm{M}} = 2(r+R)\sin(\frac{\pi}{6}-\frac{\theta_1}{2}+\frac{\theta_2}{2})-(r+R) \tag{36}$$

Assuming $\theta_1$ and $\theta_2$ are very small, we have,

$$\Delta L_{1,2}^{\mathrm{M}} = \frac{\sqrt{3}}{2}(r+R)(\theta_2-\theta_1) \tag{37}$$

Likewise, the displacements of inner and outer springs are as follows:

$$\begin{aligned}\Delta L_{1,2}^{\mathrm{I}} &= \frac{\sqrt{3}}{2}r(\theta_2-\theta_1)-\frac{\sqrt{3}}{2}R(\varphi_2-\varphi_1)\\ \Delta L_{1,2}^{\mathrm{O}} &= \frac{\sqrt{3}}{2}r(\theta_2-\theta_1)+\frac{\sqrt{3}}{2}R(\varphi_2-\varphi_1)\end{aligned} \tag{38}$$

The potential at the interface between rod 1 and rod 2 is then,

$$\begin{aligned}U_{1,2} &= \frac{1}{2}k_{1,2}^{\mathrm{I}}(\Delta L_{1,2}^{\mathrm{I}})^2+\frac{1}{2}k_{1,2}^{\mathrm{M}}(\Delta L_{1,2}^{\mathrm{M}})^2+\frac{1}{2}k_{1,2}^{\mathrm{O}}(\Delta L_{1,2}^{\mathrm{O}})^2\\ &= \frac{3}{8}\{[(1-\phi)^2k_{1,2}^{\mathrm{I}}+k_{1,2}^{\mathrm{M}}+(1+\phi)^2k_{1,2}^{\mathrm{O}}]x_{1,2}^2+(k_{1,2}^{\mathrm{I}}+k_{1,2}^{\mathrm{O}})y_{1,2}^2+2[(1+\phi)k_{1,2}^{\mathrm{O}}-(1-\phi)k_{1,2}^{\mathrm{I}}]x_{1,2}y_{1,2}\}\end{aligned} \tag{39}$$

where $k_{1,2}^{\mathrm{I}}$, $k_{1,2}^{\mathrm{M}}$ and $k_{1,2}^{\mathrm{O}}$ are the spring constants of the inner, middle and outer springs at the interface; $x_{1,2}=(r+R)(\theta_2-\theta_1)$, $y_{1,2}=R(\varphi_2-\varphi_1)$ and $\phi=\frac{R}{R+r}$.

For convenience, by defining $U_{i,i+1}=U_i, k_{i,i+1}^{\mathrm{I}}=k_i^{\mathrm{I}}, k_{i,i+1}^{\mathrm{M}}=k_i^{\mathrm{M}}, k_{i,i+1}^{\mathrm{O}}=k_i^{\mathrm{O}}$, $x_{i,(i+1)}=q_i$

and $y_{i,(i+1)} = q_{6+i}$, the potential of the interface between the *i*th rod and (*i*+1)th rod can be written as

$$U_i = \frac{3}{8}\{[(1-\phi)^2 k_i^{\mathrm{I}} + k_i^{\mathrm{M}} + (1+\phi)^2 k_i^{\mathrm{O}}]q_i^2 + (k_i^{\mathrm{I}} + k_i^{\mathrm{O}})q_{6+i}^2 + 2[(1+\phi)k_i^{\mathrm{O}} - (1-\phi)k_i^{\mathrm{I}}]q_i\, q_{6+i}\}$$

(40)

The potential of the whole system is $U = \sum_{i=1}^{6} U_i$. The matrix $\mathbf{K}$ is given by $K_{mn} = \frac{\partial^2 U}{\partial q_m \partial q_n}$, and then

$$|\mathbf{K}| = \prod_{i=1}^{6} a_i + \prod_{i=1}^{6} b_i \tag{41}$$

where

$$a_i = \frac{9}{16}[(1-\phi)^2 (k_i^{\mathrm{I}})^2 + (1+\phi)^2 (k_i^{\mathrm{O}})^2 + 2(1+\phi^2)k_i^I k_i^{\mathrm{O}} + k_i^{\mathrm{M}}(k_i^{\mathrm{I}} + k_i^{\mathrm{O}})] \tag{42}$$

$$b_i = \frac{9}{16}[(1-\phi)k_i^{\mathrm{I}} - (1+\phi)k_i^{\mathrm{O}}]^2 \tag{43}$$

According to Eq. 35, the free energy of activation of phosphorylation or dephosphorylation at one interface is

$$\begin{aligned}\Delta G^{\neq} &= G^{\neq} - G \\ &= \frac{1}{2}k_{\mathrm{B}}T \ln\frac{|\mathbf{K}^{\neq}|}{|\mathbf{K}|} = \frac{1}{2}k_B T \ln\frac{\prod_{i=1}^{6} a_i^{\neq} + \prod_{i=1}^{6} b_i^{\neq}}{\prod_{i=1}^{6} a_i + \prod_{i=1}^{6} b_i} \\ &\approx \frac{1}{2}k_{\mathrm{B}}T\left(\sum_{i=1}^{6}\ln\frac{a_i^{\neq}}{a_i} + \prod_{i=1}^{6}\frac{b_i^{\neq}}{a_i^{\neq}} - \prod_{i=1}^{6}\frac{b_i}{a_i}\right),\end{aligned} \tag{44}$$

provided that $\prod_{i=1}^{6} a_i >> \prod_{i=1}^{6} b_i$, and $\prod_{i=1}^{6} a_i^{\neq} >> \prod_{i=1}^{6} b_i^{\neq}$.

Note that in $a_i^{\neq}$ (or $b_i^{\neq}$) not all the spring constants vary greatly, only the ones (or one) related to the reaction interface change the most. Because we do not know the detailed relationship between the three spring constants in ground state and those in

transition state, two hypothetical correlations are made as, $a_i^{\neq} = f_i a_i$, $b_i^{\neq} = g_i b_i$, where $f_i$ and $g_i$ are in general the functions of the corresponding spring constants. Thus, one can have

$$\Delta G^{\neq} = \frac{1}{2} k_{\mathrm{B}} T (P + Q \prod_{i=1}^{6} \frac{b_i}{a_i}) \tag{45}$$

where

$$P = \sum_{i=1}^{6} \ln f_i \tag{46}$$

$$Q = -1 + \prod_{i=1}^{6} \frac{g_i}{f_i} \tag{47}$$

Simply, let

$$\frac{b_i}{a_i} = 1 - \Lambda_i \tag{48}$$

where

$$\Lambda_i = \frac{4k_i^{\mathrm{I}} k_i^{\mathrm{O}} + k_i^{\mathrm{M}} (k_i^{\mathrm{I}} + k_i^{\mathrm{O}})}{[(1-\phi)k_i^{\mathrm{I}} - (1+\phi)k_i^{\mathrm{O}}]^2 + 4k_i^{\mathrm{I}} k_i^{\mathrm{O}} + k_i^{\mathrm{M}} (k_i^{\mathrm{I}} + k_i^{\mathrm{O}})} \tag{49}$$

Expanding $\prod_{i=1}^{6} (1 - \Lambda_i)$, we finally have

$$\begin{aligned} \Delta G^{\neq} = \frac{1}{2} k_{\mathrm{B}} T [P + Q(1 - \sum_{i=1}^{6} \Lambda_i + \sum_{i<j} \Lambda_i \Lambda_j - \sum_{i<j<k} \Lambda_i \Lambda_j \Lambda_k \\ + \sum_{i<j<k<l} \Lambda_i \Lambda_j \Lambda_k \Lambda_l - \sum_{i<j<k<l<m} \Lambda_i \Lambda_j \Lambda_k \Lambda_l \Lambda_m + \Lambda_1 \Lambda_2 \Lambda_3 \Lambda_4 \Lambda_5 \Lambda_6)] \end{aligned} \tag{50}$$

The expression of free energy of activation in Eq. 50 contains $n$-interface mode interaction (as in the round bracket), where $n$=1 to 6. $P$ and $Q$ reflect the effects caused by the difference between transition state and ground state of KaiC hexamer (according to the definition of $f_i$ and $g_i$), especially at the reaction interface. Because the differences between transition state and ground state at the other non-reaction

interfaces are much less, $P$ and $Q$ mainly related to the changes in the reaction-interface, particularly for $P$. Thus, the term $\frac{1}{2}k_{\mathrm{B}}T(P+Q)$ in Eq. 50 corresponds to $\Delta G_{\mathrm{p}}^{\neq *}+\Delta\Delta E_{\mathrm{p}}^{\neq}$ in Eq. 24.

If $f_i = g_i$, then $Q = 0$, hence the terms in the round bracket in Eq. 50 are vanished, and the free energy obtained can not bring about kinetic cooperativity in KaiC phosphorylation or dephosphorylation. The necessary condition of generating cooperativity is $f_i \neq g_i$, which means the alterations of the three types of spring constants have to be different at transition state. Thus, it suggests that the kinetic cooperativity is inherent in the asymmetrical distribution of flexibility at each KaiC interface. According to Eq. 49, one can have $0 < \Lambda_i < 1$. If $f_i > g_i$, then $-1 < Q < 0$, and in this condition, the terms for multi-interface interactions are also diminished. Therefore, we infer $f_i < g_i$ (the alternation of $a_i$ is less than that of $b_i$), which suggests that at transition state the middle spring (taking effect in $a_i$) attenuates the effects of changes in the outer and inner springs. Probably, the middle spring is mainly responsible for keeping the integrity and stability of the KaiC hexamer.

$\Lambda_i$ in Eq. 49 characterizes the contribution of certain interface in the multi-interface interaction at ground state, and is a structure-based factor. The quantity of $\Lambda_i$ is strictly restrained, otherwise the variation in the free energy for different phosphorylation state could be extremely large, which leads the Kai system unstable. The terms with $\Lambda_i$ in Eq. 50 have both positive and negative components, which technically hinders the further analysis in the free energy changes.

Actually, different time scales exist among the multi-interface interaction modes

represented in the round bracket in Eq. 50, the 1- or 2-interface interaction belongs to the fast modes, whereas the 4-, 5- and 6-interface interaction modes are relatively slow. In particular, the 3-interface (triplet) interaction may contain both fast and slow modes. Thus, we can split the 3-interface interaction mode into two parts, one for fast modes and one for slow modes, and make a coarse redistribution in the free energy. Two general levels of the free energy are defined as the quasi-local mode ($X_{\mathrm{QL}}$) and the global mode ($X_{\mathrm{G}}$), characterized by:

$$X_{\mathrm{QL}} = -\sum_{i=1}^{6}\Lambda_i + \sum_{i<j}\Lambda_i\Lambda_j - \varepsilon\sum_{i<j<k}\Lambda_i\Lambda_j\Lambda_k = -\eta\lambda_{00}^{a1}\lambda_{01}^{a2}\lambda_{10}^{a3}\lambda_{11}^{a4} \tag{51}$$

$$\begin{aligned} X_{\mathrm{G}} = &-(1-\varepsilon)\sum_{i<j<k}\Lambda_i\Lambda_j\Lambda_k + \sum_{i<j<k<l}\Lambda_i\Lambda_j\Lambda_k\Lambda_l \\ &- \sum_{i<j<k<l<m}\Lambda_i\Lambda_j\Lambda_k\Lambda_l\Lambda_m + \Lambda_1\Lambda_2\Lambda_3\Lambda_4\Lambda_5\Lambda_6 = -\kappa\lambda_{00}^{b1}\lambda_{01}^{b2}\lambda_{10}^{b3}\lambda_{11}^{b4} \end{aligned} \tag{52}$$

We introduce the apparent interaction factor $\lambda_{mn}$ ($m$, $n$=0, 1, the same as in Eq. 24) to approximate the combined effects of $\Lambda_i$. Eq. 51 and Eq. 52 stand for the quasi-local mode and the global mode, respectively, where $\sum_{ai=1}^{4} ai = 3$ and $\sum_{bi=1}^{4} bi = 6$. $\eta$ and $\kappa$ are the corresponding coefficients, both positive. $\varepsilon$ is the fraction of 3-interface interaction in the fast modes, while $1-\varepsilon$ in the slow mode, $0 < \varepsilon < 1$.

Further analysis shows that the appropriate values of $\varepsilon$ and $\Lambda$ are down to narrow ranges. In such conditions, the free energy of activation in Eq. 50 can be safely approximated by the coarse hierarchical decomposition method as in Eq. 24. The further analysis can be found in section 1.2.5 below.

### 1.2.5 Estimation of parameters

Our model contains a number of dimensions and parameters. There are 88 basic parameters, as mentioned in section 1.2.1. The values of these parameters must be chosen reasonably and logically so that they can be self-consistent, and also reproduce as many as possible the existing experimental data. To fulfill the latter requirement, a multi-objective optimization seems to be a more systematic method for the estimation of parameters. In fact, various experimental results are equally important objectives for the optimization, such as:

(1) distribution of KaiC phosphoforms in steady state dynamics for binary interactions;

(2) small effect of KaiB on the KaiC phosphorylation;

(3) amplitude, period, and most importantly the waveform or phase distribution of overall KaiC phosphorylation cycle (the duration of phosphorylation phase is shorter than that of the dephosphorylation phase);

(4) amplitude and phase differences between T432 ($Y_T$) and S431 ($Y_S$) phosphorylation;

(5) diverse waveforms and phase relationships of hexameric KaiC phosphoforms;

(6) temperature compensation;

(7) relatively weak dependence of KaiA-KaiC complex formation on KaiC phosphorylation state;

(8) relatively strong dependence of KaiB-KaiC binding on KaiC phosphorylation state, especially on S431 phosphorylation level, and so forth.

The multi-objective approach in this case adds a great amount of complexity in the

global optimization process. In this work, we do not use this systematic method to determine the values of the parameters. Instead, we manually tune the parameters in two major steps. First, the 88 parameters are not fully independent, so a qualitative and semi-quantitative method is used to analyze the boundaries and constraints of the parameters. Second, the final values of the parameters are determined by fitting various experimental data, which is under the limit of the first step analysis.

(i) Qualitative and semi-quantitative analysis

There are many sets of kinetic constants in the model, yet they all follow a similar way of estimation. For example, in Eq. 24,

$$\Delta G^{\neq}_{00\mathrm{Tp}} = \Delta G^{\neq *}_{\mathrm{p}} + \Delta\Delta E^{\neq}_{\mathrm{p}}[1 + \sigma^{\neq}_{00\mathrm{Tp}}\lambda^{i}_{00}\lambda^{j}_{01}\lambda^{k}_{10}\lambda^{l}_{11} + \omega\sigma^{\neq}_{00\mathrm{Tp}}\lambda^{\alpha-1}_{00}\lambda^{\beta}_{01}\lambda^{\gamma}_{10}\lambda^{\delta}_{11}]$$

$\Delta G^{\neq *}_{\mathrm{p}}$ (the elementary free energy of activation for an isolated phosphorylation site), is assumed to be 25 $k_{\mathrm{B}}T$, a value that falls in the range of typical free energy for general Thr/Ser phosphorylation. The other parts of the right hand side in Eq. 24 represent the free energy due to intra- and inter-subunit interactions in KaiC hexamer. According to the hierarchical decomposition of the free energy, we estimate that the value of the local mode energy $\Delta\Delta E^{\neq}_{\mathrm{p}}$ is around -12 $k_{\mathrm{B}}T$ which approximately equals to the free energy released from hydrolysis of one ATP molecule. It means that besides one ATP being used as a substrate for phosphorylation, hydrolysis of at least one extra ATP is needed to drive the conformational change at the interface so that each KaiC hexamer can approach the transition state.

The term $\sigma^{\neq}_{00\mathrm{Tp}}\lambda^{i}_{00}\lambda^{j}_{01}\lambda^{k}_{10}\lambda^{l}_{11}$ in the square bracket is the factor of the relative small part for the quasi-local mode energy, and it must be less than 1. $\omega\sigma^{\neq}_{00\mathrm{Tp}}\lambda^{\alpha-1}_{00}\lambda^{\beta}_{01}\lambda^{\gamma}_{10}\lambda^{\delta}_{11}$

represents the smallest portion, the global mode free energy. $\omega$ is the scaling factor of the smallest energy portion, thus it must satisfy $0<\omega<1$ and $\sigma^{\neq}_{00\mathrm{Tp}}\lambda^{i}_{00}\lambda^{j}_{01}\lambda^{k}_{10}\lambda^{l}_{11} > \omega\sigma^{\neq}_{00\mathrm{Tp}}\lambda^{\alpha-1}_{00}\lambda^{\beta}_{01}\lambda^{\gamma}_{10}\lambda^{\delta}_{11}$. The parameter $\lambda$ measures one certain monomer's ($m_{00}$, $m_{01}$, $m_{10}$ or $m_{11}$) influence on KaiC phosphorylation as it participates in the intersubunit interaction. We assign the impact of $m_{00}$ as a standard by setting $\lambda_{00}=1$. It means that if S431 and T432 all are empty, $m_{00}$ has basal effect on the phosphorylation of S431 or T432 in KaiC hexamer. Experiments show that T432 positively affect phosphorylation, while S431 acts more like an attenuator [10,11]. The monomer $m_{10}$, where T432 is phosphorylated (or occupied), it strengthens the phosphorylation rate in KaiC hexamer. If S431 is occupied (for $m_{01}$), the phosphorylation rate in KaiC hexamer is attenuated. Therefore, we set $\lambda_{10}\geq 1$ (for $m_{10}$) and $\lambda_{01}\leq 1$ ($m_{01}$). The equal signs in the expressions represent the case in which the phosphorylation state at S431 or T432 has a very weak effect on regulating KaiC phosphorylation (or dephosphorylation). As to $m_{11}$ where both T432 and S431 are occupied, we hypothesize that the effects of both sites may be partially counterbalanced. If T432 slightly prevails, then we have $\lambda_{00}\leq\lambda_{11}\leq\lambda_{10}$, thus $\lambda_{01}\leq\lambda_{00}\leq\lambda_{11}\leq\lambda_{10}$. If S431 is favored, then $\lambda_{01}\leq\lambda_{11}\leq\lambda_{00}$, and finally $\lambda_{01}\leq\lambda_{11}\leq\lambda_{00}\leq\lambda_{10}$. $\sigma^{\neq}$ represents the effect of the intra-subunit interaction on its own phosphorylation rate at the transition interface. The relationship of this parameter's magnitude is designed similar to that of $\lambda$. The estimations of parameters for the free energies of dephosphorylation and Kai protein interactions run in the same vein.

Furthermore, the simple normal mode analysis in section 1.2.4 can also give a quantitative estimation of constraints for some parameters.

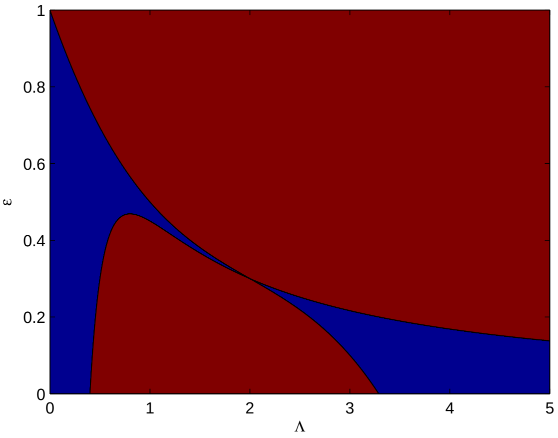


Fig. S4. The constraints on the values of *ε* and *Λ* based on Eq. 53-54. The parameter values in the blue area of *ε*-*Λ* space fulfill the requirement of both $X_{QL}$<0 and $X_G$<0. The values of *ε* and *Λ* should be taken only within the blue area.

According to Eq. 51 and Eq. 52, to roughly estimate the approximate quantities of $\varepsilon$, $\Lambda_i$ and $\lambda_{mn}$, we can assume that $\Lambda_i$ and $\lambda_{mn}$ take certain fixed values, e.g. $\Lambda$ and $\lambda$, considering $\Lambda_i$ and $\lambda_{mn}$ change very slightly (under restrict constraints). Then, Eq. 51 and Eq. 52 can be reduced to:

$$X_{\mathrm{QL}} = -6\Lambda + 15\Lambda^2 - 20\varepsilon\Lambda^3 = -\eta\lambda^3 \qquad (53)$$

$$X_{\mathrm{G}} = -20(1-\varepsilon)\Lambda^3 + 15\Lambda^4 - 6\Lambda^5 + \Lambda^6 = -\kappa\lambda^6 \qquad (54)$$

Considering the simplest situation, to keep $X_{\mathrm{QL}} < 0$, $X_{\mathrm{G}} < 0$ (for temperature compensation) and $|X_{\mathrm{QL}}| > |X_{\mathrm{G}}|$ (hierarchical energy distribution), values of *ε* and *Λ* have a narrow range under the quantitative analysis. To satisfy the above requirements for both $X_{\mathrm{QL}}$ and $X_{\mathrm{G}}$, the values of *ε* and *Λ* have to be constrained in the blue area in Fig. S4. Additionally, the value of *Λ* need to be close to 1, otherwise the energy differences among KaiC phosphorylation states are too large, which is mainly due to the product of multiple *Λ* in Eq. 51-54. Therefore, we found a appropriate range for *ε*

and $\Lambda$, i.e. approximately, $\varepsilon \sim 0.5$, $0.8 \le \Lambda \le 1.5$. Then, $\eta^2/\kappa$ is narrow down to a certain range. The quantity of $\lambda$ can be coordinated with the absolute value of $\eta$ and $\kappa$, as well as $P$ and $Q$ to finally make the value of the full free energy reasonably. Because $P$ and $Q$ are restrained by the temperature compensation effect, the value of $\lambda$ is in fact down to a narrow range as well, which set up a strict constraint for the parameter estimation.

(ii) Fitting the parameters to experimental data

In fact, the qualitative and semi-quantitative estimations in section 1.2.1 only set some boundaries and constraints on the parameters, yet the actual values of the parameters need to be carefully calculated so that the final dynamic results of Kai oscillator can be consistent with a series of experiments. Besides the properties of KaiC phosphorylation oscillation, we use three additional aspects of experimental facts to fit the parameters.

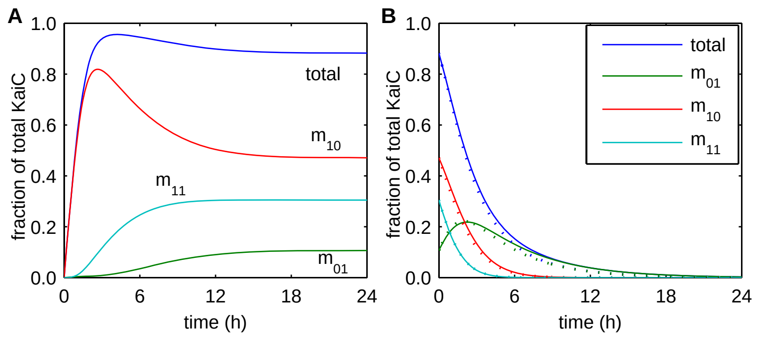


**Fig. S5.** Steady state dynamics of monomeric KaiC phosphoforms ($m_{01}$, $m_{10}$ and $m_{11}$). (A) KaiC phosphorylation in the presence of KaiA. (B) KaiC dephosphorylation in the absence (solid lines) and in the presence (dotted lines) of KaiB. The notion "total" in A and B denotes $m_{01}$+$m_{10}$+$m_{11}$.

(1) The distribution of monomeric KaiC phosphoforms ($m_{01}$, $m_{10}$, $m_{11}$) at steady state under the binary interactions of KaiA and KaiC. According to the work by Nishiwaki et al. [12], when KaiC was incubated with KaiA for 8 hours, >90% of KaiC

were converted to phosphorylated forms. The fraction of $m_{10}$, $m_{01}$ and $m_{11}$ are 46.6%, 11.9% and 33%, respectively. The parameters in our model are designed to reproduce the similar result, as shown in Fig. S5A.

(2) The dephosphorylation dynamics of highly phosphorylated KaiC where KaiA and KaiB are absent. The temporal profiles of free KaiC dephosphorylation are shown in Fig. S5B (solid lines), which can be compared with Ref. [1] and Ref. [11].

(3) The dephosphorylation patterns of KaiC in the presence of KaiB. There is no substantial difference between the profiles of KaiC dephosphorylation with and without KaiB. However, the two profiles are slightly different, as reported by Rust et al. [11] in their Fig. S2A. The profiles of KaiC dephosphorylation with KaiB in our model are just in the similar case, referring to the dotted lines in Fig. S5B.

## 2. Additional results

### 2.1 Functional differentiation between S431 and T432

Our simulation results suggest that T432 is a major amplitude regulator, while S431 the major phase regulator. Further, we provide a simple method to prove this argument. The strongest interaction between S431 and T432 is the intra-subunit interaction at the transition interface, so we simply modify the corresponding harmonic factors and check the result. First, we change the strength of phosphorylated T432 affecting S431 (both in phosphorylation and dephosphorylation states) by multiplying a factor $f_T$ to the parameters $\sigma^{\neq}_{10\mathrm{Sp}}(\mathrm{AC})$, $\sigma^{\neq}_{10\mathrm{Sp}}(\mathrm{ABC})$, $\sigma^{\neq}_{11\mathrm{Sdp}}(\mathrm{C})$, $\sigma^{\neq}_{11\mathrm{Sdp}}(\mathrm{BC})$ and $\sigma^{\neq}_{11\mathrm{Sdp}}(\mathrm{ABC})$, where the notions in the bracket represent which KaiC

complexes the parameters belong to (refer to Table S5). As shown in Fig. S6A, the amplitude of the total phosphorylation changes remarkably. In the similar way, we check the result by changing the strength of the effect of phosphorylated S431 on T432. By multiplying a factor $f_S$ to the parameters $\sigma^{\neq}_{01Tp}(AC)$, $\sigma^{\neq}_{01Tp}(ABC)$, $\sigma^{\neq}_{11Tdp}(C)$, $\sigma^{\neq}_{11Tdp}(BC)$ and $\sigma^{\neq}_{11Tdp}(ABC)$, we obtain the results as in Fig. S6B. The amplitude has slightly changed comparing to that in Fig. S6A, but the phase of the phosphorylation peak has shifted considerably. As $f_S$ increases, the ratio of the duration of phosphorylation phase and that of dephosphorylation phase decreases from ~0.77 to ~0.68, while the ratio for changing $f_T$ varies only from ~0.73 to ~0.75.

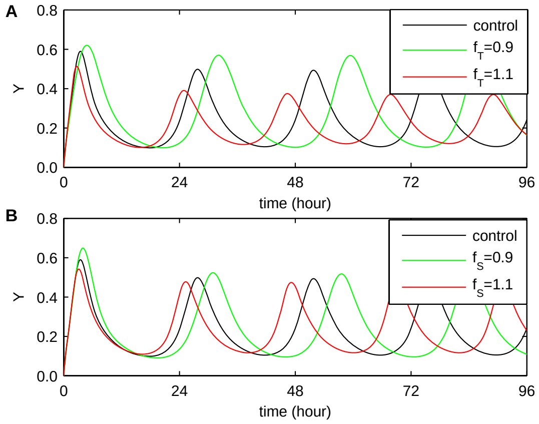


**Fig. S6.** Simple demonstration for the functional differentiation of S431 and T432. (A) Changing the strength of the effect of T432 on S431 phosphorylation and dephosphorylation at the transition interface. The factor $f_T$ is multiplied to $\sigma^{\neq}_{10Sp}(AC)$, $\sigma^{\neq}_{10Sp}(ABC)$, $\sigma^{\neq}_{11Sdp}(C)$, $\sigma^{\neq}_{11Sdp}(BC)$, $\sigma^{\neq}_{11Sdp}(ABC)$ and, where the notions in the bracket represent which the KaiC complexes the parameters belong to. (B) Changing the strength of the effect of S431 on T432 phosphorylation and dephosphorylation at the transition interface. The factor $f_S$ is the similar scaling factor as $f_T$.

## 2.2 Stochastic simulation of the Kai reaction network

Stochastic simulation is performed based on the full Kai reaction network to specifically mimic the *in vivo* KaiC phosphorylation dynamic. Here, associations-dissociations of KaiA-KaiC and KaiA-KaiBC are described by general

forward and backward reactions rather than rapid equilibrium. The total molecule numbers of Kai proteins are chosen as: 2000 KaiA dimers, 6034 KaiB dimers and 2000 KaiC hexamers. The effective volume of one single cyanobacterial cell is set to be $5.73\times10^{-15}$ L. The stochastic simulation is performed using Gillespie algorithm [13]. Similar results are obtained as those in deterministic model, shown in Fig. S7.

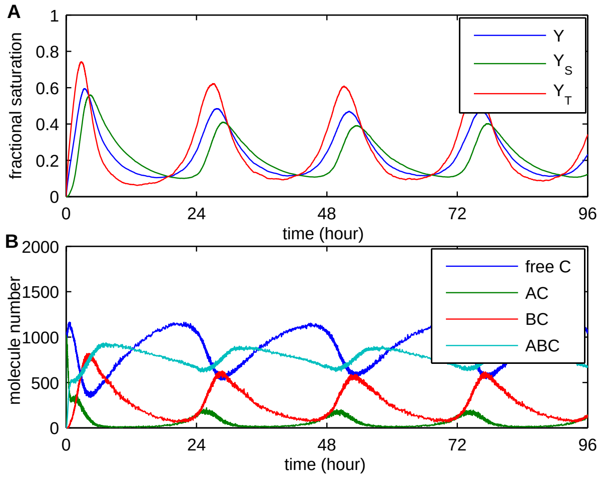


**Fig. S7.** Stochastic simulation of the full model of Kai oscillator. (A) Circadian oscillations of $Y$ (blue), $Y_S$ (green) and $Y_T$ (red), defined as in the deterministic model. (B) Oscillations of free KaiC (blue), KaiAC (green), KaiBC (red) and KaiABC (cyan). The total molecule number of KaiC hexamers is 2000.

## 2.3 Diverse circadian patterns of KaiC phosphoforms

Fig. S8 shows various temporal profiles of hexameric KaiC phosphoforms obtained by stochastic simulation. We have categorized the waveforms of KaiC into four groups (in main text), but the waveforms are actually quite variable, even for the same group.

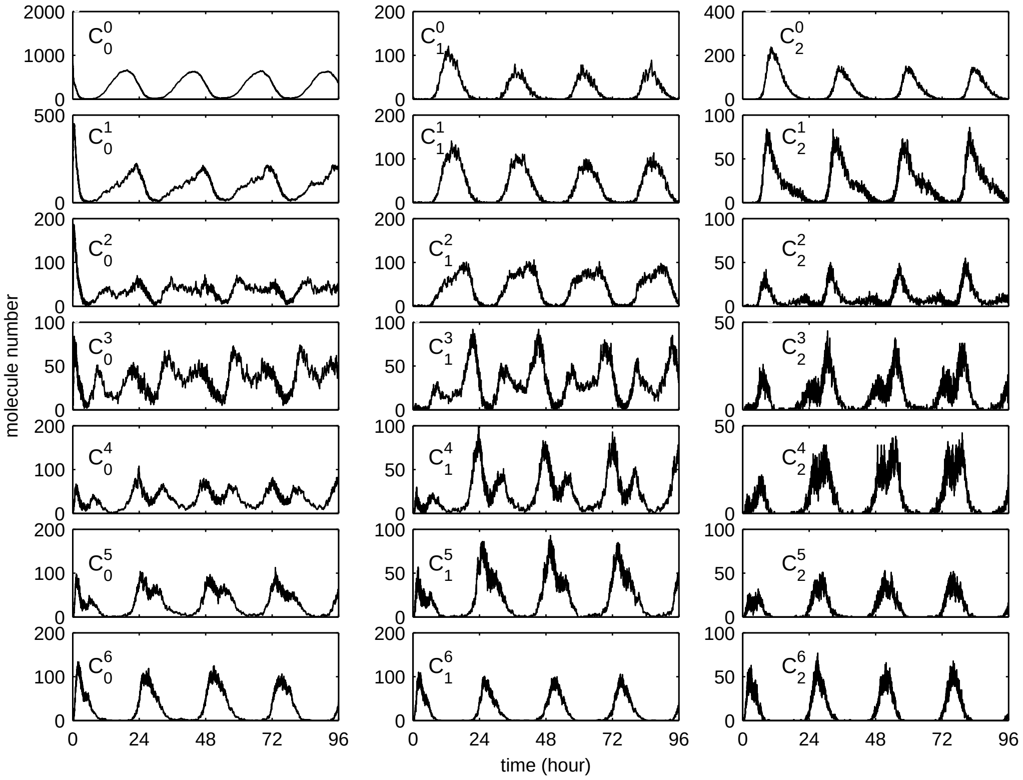


**Fig. S8.** Diverse waveforms of hexameric KaiC phosphoforms. The result is obtained by stochastic simulation. 21 KaiC phosphoforms are shown: $C_0^0 \rightarrow C_0^6$ (left column), $C_1^0 \rightarrow C_1^6$ (middle column) and $C_2^0 \rightarrow C_2^6$ (right column).

## 2.4 Temperature compensation in Kai system

The wild-type Kai clock is extremely robust against the temperature fluctuation, with a $Q_{10}$ factor ~1.1 or even less [14,15]. It suggests that a low apparent free energy of activation for phosphorylation or dephosphorylation at ambient temperature. The estimation shows that a relatively large enough local mode free energy (~12 $k_B T$) considerably reduces the intrinsic free energy of activation (details in Table S5). A $Q_{10}$ factor of 1.06 is obtained in our model (Fig. S9A). In essence, the robust temperature compensation is inherent to the stability of the fundamental structure of KaiC hexamer. We further found that, to maintain strong robustness, the formation of KaiAC and KaiBC should take place spontaneously ($\Delta G<0$), whereas the formation of KaiABC

requires extra energy supply (ΔG>0).

Our model reproduces the self-temperature-compensation in phosphorylation and dephosphorylation [14], shown in Fig. S9B-C. This is so due to the low apparent free energy of activation. The local mode free energy mostly responsible for the integrity and stability of KaiC hexamer plays the main part in reducing the high intrinsic free energy of activation.

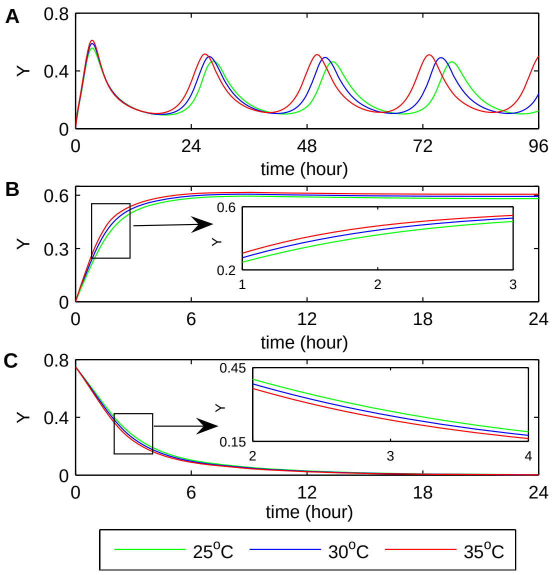


**Fig. S9.** Temperature compensation in Kai system. (A) Temperature compensation of the KaiC phosphorylation oscillation. From 25°C (green) to 35°C (red), the period decreases ~1.5 h. (A) In the absence of KaiB, KaiA stimulates KaiC phosphorylation at 25°C (green), 30°C (blue) and 35°C (red). (B) Same temperature tests for dephosphorylation of KaiC with neither KaiA nor KaiB.

## 2.5 Bifurcation diagrams for variations in concentration of KaiA and KaiB

Variations in protein concentrations affect the Kai oscillator's dynamic properties. As shown in Fig. S10A, a supercritical Hopf bifurcation occurs at $[A]_T/[A]_T^+ = 0.52$ with a period of 23.5 h (Fig. S10B). $[A]_T^+$ (or $[B]_T^+$ later mentioned) is the standard KaiA (or KaiB) concentration in ref [1]. The period tends to first increase then decrease gradually with the increase in the ratio of $[A]_T/[A]_T^+$ till the oscillation

disappears at the second Hopf bifurcation point where $[A]_T/[A]_T^+$ is ~1.9. The amplitude attains the maximum at $[A]_T/[A]_T^+ \approx 1.0$, then decreases slowly up to the second Hopf point.

For $[B]_T/[B]_T^+$, the oscillation occurs when this ratio is approximately greater than 0.32 (Fig. S10C). The amplitude and the period slightly decrease as $[B]_T/[B]_T^+$ increases (Fig. S10C and Fig. S10D). The sustained oscillation still holds true up to $[B]_T/[B]_T^+ = 8.0$ where the period is ~21.1 h. The similar results can be found in previous experiments [1] and theoretical simulations [16]. The bifurcation analysis is performed with XPPAUT software package [17].

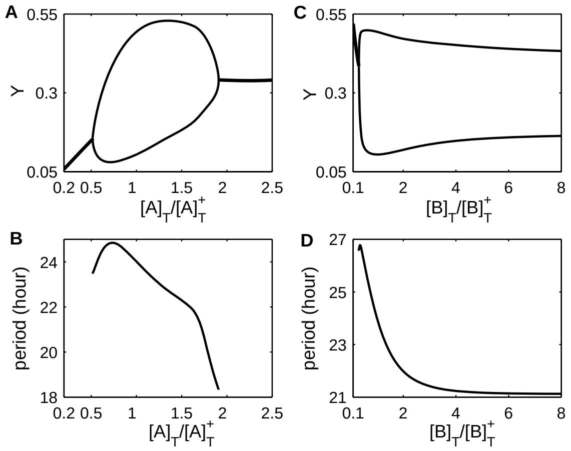


**Fig. S10.** Dependence of oscillation amplitude and period on the total concentrations of KaiA and KaiB. (A) Phosphorylation amplitude varies with $[A]_T/[A]_T^+$, where $[A]_T^+$ (or $[B]_T^+$ later mentioned) stands for the standard total concentration of KaiA (or KaiB). Two Hopf bifurcation points are found. Oscillation occurs within the range of $0.52 \leq [A]_T/[A]_T^+ \leq 1.9$. (B) Period dependence on $[A]_T/[A]_T^+$. (C) $[B]_T/[B]_T^+$ variation shows only one Hopf bifurcation point $[B]_T/[B]_T^+=0.32$. The amplitude of oscillation is slightly changed as $[B]_T/[B]_T^+$ changes in a wide range. (D) Period dependence on $[B]_T/[B]_T^+$.

## 2.6 Dynamics under concerted changes in Kai protein concentrations

The Fig. 7C and D in the main text have shown the robustness of the oscillator

against the concerted variations in Kai proteins' concentrations. According to our simulations, the period decreases from 24 h to 21 h as the concerted protein concentrations increase from 1× to 5×, and the amplitude only changes slightly. So far, we are unable to fully simulate the results observed by Kageyama et al. [1], where the increase in concentrations has nearly no effect on the length of the period. The tendency of the period change in our Fig. 7C seems to be consistent with the work by Kageyama et al. [1], where they found that lowering the protein concentration lengthens the period of Kai oscillator.

On the other hand, a more recent work by Rust et al. [11] showed an opposite tendency in which the period is shortened as the protein concentrations decrease (from 4× to 0.5×, details see their Figure S8A [11]). However, the common point of the two works by Kageyama et al. and Rust et al. is that the period variation is quite small as the total Kai protein concentrations increase even several folds.

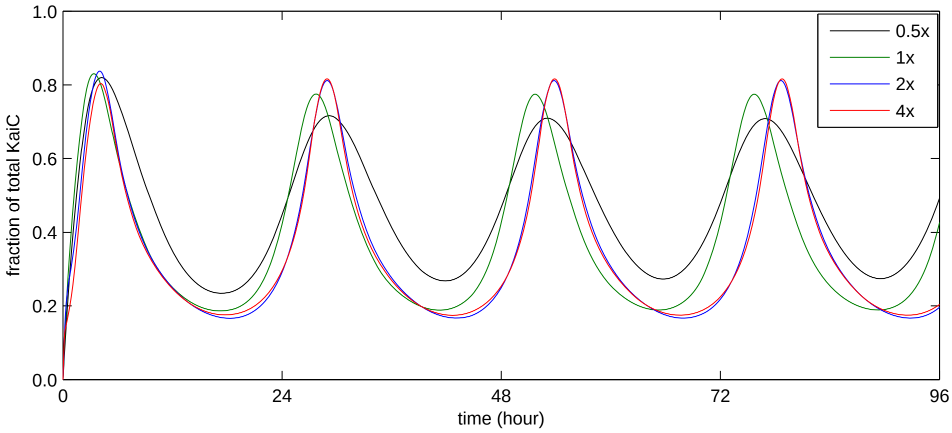


Fig. S11. KaiC phosphorylation under concerted changes in Kai protein concentrations. The concentrations used in the simulation are: 0.5×, 1×, 2×and 4× standard Kai protein concentrations.

Particularly, we have adjusted our model (parameters see Table S4 and S5) and

obtained the same tendency of period variation (with protein concentrations) as the results shown by Rust et al. [11]. In the original model for mimicking the experiments by Kageyama et al., we set $kb_0^0$ = $kb_0^1$ = 0, i.e. the forward reactions of KaiB binding to free $C_0^0$ and $C_0^1$ (KaiC hexamer with one T432 phosphorylated) are not allowed, whereas we let free $C_0^1$ be able to bind KaiB and only leave $kb_0^0$ = 0 in the newly adjusted model. Based on this change, we tune the other parameters as slightly as possible so that it can mostly reproduce the results made by Rust et al. [11], and keep all our earlier simulation results valid. Fig. S11 shows the new result of dynamic robustness of Kai oscillator against changes in total protein concentrations. The periods of the four samples are 23.83 h (0.5×), 23.99 h (1×), 24.85 h (2×) and 24.92 h (4×), respectively.

**2.7 Synchronization of different phased Kai samples**

We performed several additional mixing simulations using samples at different phase points. Particularly, Fig. 7B (in the main text) reveals that approximately three situations could occur after mixing. Here, we explicitly reproduce these behaviors in Fig. S12. The mixing time point is set to be the zero time in Fig. S12. Two profiles rapidly enter the phosphorylation (red) and dephosphorylation (blue) phase as soon as the samples are mixed (note the initial slope of the profiles), respectively, while the third profile (black) changes its rate much slowly and finally enters the dephosphorylation phase.

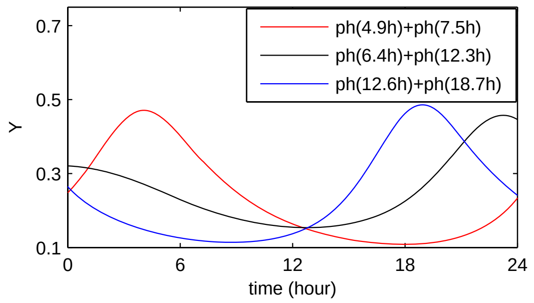


**Fig. S12.** Perturbation of KaiC phosphorylation by mixing samples in different phases. Three cases of mixing are performed: rapid shift to phosphorylation phase (red), rapid shift to dephosphorylation phase (blue) and slowly synchrony into dephosphorylation phase. Ph (T) represents a sample with the phase T (hour) at mixing time. All the mixing times are set to be zero.

## 2.8 Simulation of mixing Kai samples with non-standard concentrations

We mixed two equal-amount Kai samples with non-standard concentrations, and keep the final mixture to be standard. The non-standard Kai systems are prepared by changing the standard concentration of either KaiA or KaiB. Specifically, we first set up two samples with 0.7× and 1.3× standard KaiA, respectively, leaving KaiB and KaiC standard. The two systems have different periods, ~26.42 h for 0.7× KaiA and ~22.42 h for 1.3×. The simulation started with mixing the two samples at both their peaking points, and the mixing was performed every 12 hours. What we trace is the time span (starting from the mixing time) for the system to return to its normal oscillation state. Generally, it takes 1 ~ 2 days to return to the normal Kai oscillation state, as shown in Fig. S13A. The recovery time changes quasi-periodically with a period of ~ 1 week. To obtain an absolute periodic rhythm, the ratio of the two samples' periods must be a rational number, i.e. it can be expressed as the quotient $a/b$ of two positive integers. In fact, this relation may not be fully satisfied all the time. Even if it fulfills the requirement, the final period of recovery time could be too large

to observe because of two large primes *a* and *b*. It usually (if lucky) acts in a pseudo-periodic way shown in Fig. S13A.

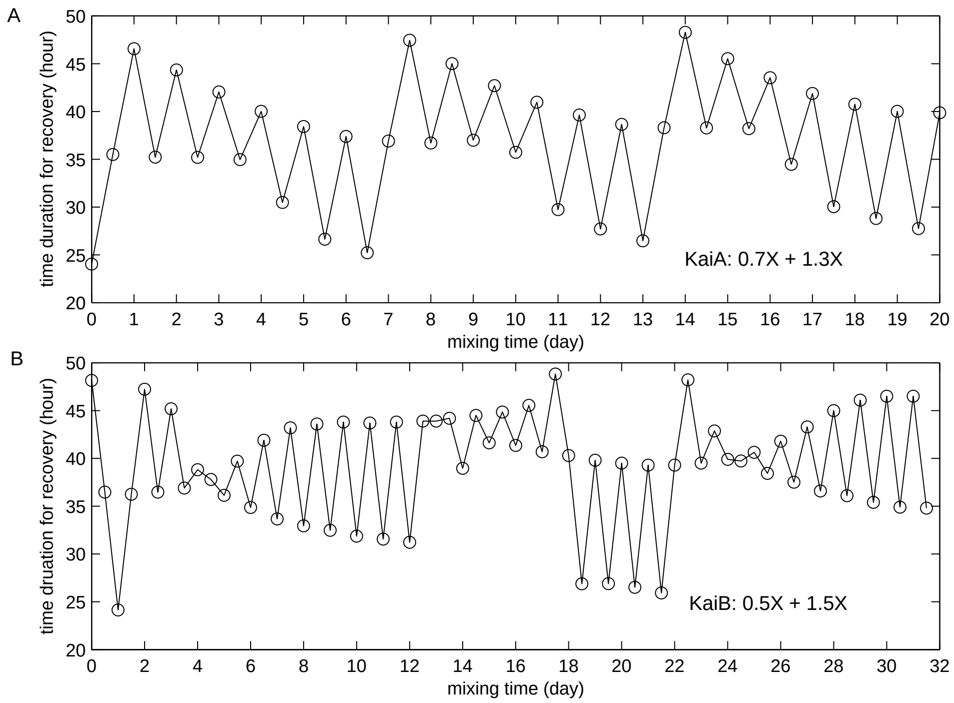


Fig. S13. The recovery time as a function of mixing time of two non-standard Kai samples. (A) The concentrations of KaiA for the two samples are 0.7× and 1.3× standard KaiA, respectively, while those for KaiB and KaiC remains standard. (B) The concentrations of KaiB are 0.5× and 1.5× standard KaiB for the two samples, respectively, while those for KaiA and KaiC remains standard. In both (A) and (B), the initial time of mixing is the peaking time for both samples. Parameter set used is the new one obtained in section 2.6.

We performed the similar simulation with non-standard KaiB (Fig. S13B). Here, we use 0.5× (period: ~ 22.42 h) and 1.5× (period: ~ 24.77 h) KaiB samples (KaiA and KaiC keep standard). The profile of recovery time seems not to be periodic compared with that in the non-standard KaiA case. The period cannot be observed at least within ~ 32 days, but a pseudo-periodic profile may be observable for a long time span.

Based on the results analyzed above, we may guess that a population of cyanobacteria could make use of this mechanism to produce non-circadian rhythms

with period longer than a day (e.g. a week or a month), if the strict periodicity is not required. The biological significance of this effect is not clear at present.

### 2.9 Reduced model and monomer shuffling

KaiC reaction network exhibits asymmetry dynamic properties due to the differentiation of S431 and T432, thus a reduced model can approximately describe the full model dynamics. For the reduced reaction network, we remove the nodes with $s \geq 4$ in the full network. Using the same parameters in the full model, the period of KaiC phosphorylation in the reduced model is a little longer than that in the full model (period ~26 h).

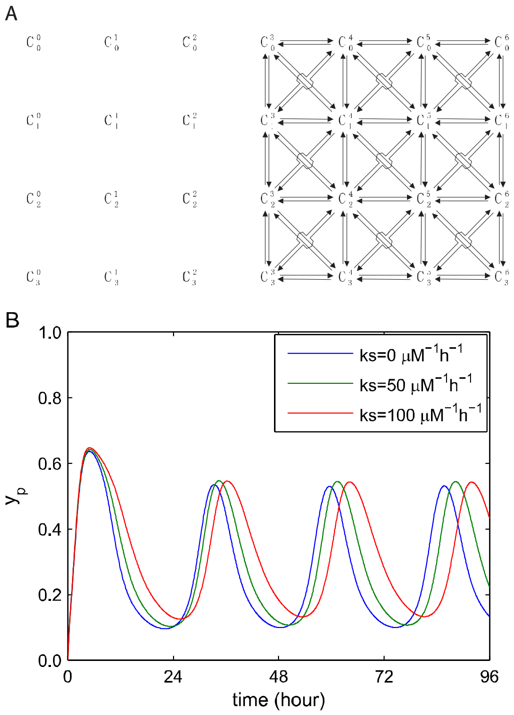


**Fig. S14.** Simulation of KaiC monomer shuffling. (A) Network for monomer shuffling based on the reduced model. Only nodes with $t \geq 3$ are assumed to be able to exchange monomers. The monomer shuffling reaction directions of some nodes are parallel to those of KaiC phosphorylation-dephosphorylation, while others are along the diagonal directions of a square element unit in the network. (B) Oscillatory KaiC phosphorylation with monomer shuffling in the

reduced model. The increase of the shuffling rate *ks* results in longer period and smaller amplitude in KaiC phosphorylation.

Based on the reduced model, we performed the monomer shuffling simulation. Only the free KaiC hexamers with $t \geq 3$ are assumed to be able to lose and gain monomers. The basic rule of monomer shuffling is to exchange only one KaiC monomer at one time, not involving exchange of dimers or higher ordered oligomers. As drawn in Fig. S14A, $\mathrm{C}_s^t$ can only become one of its nearest nodes after a single shuffling. $\mathrm{C}_s^t$ has maximum eight nearest nodes, four lie in the phosphorylation-dephosphorylation direction and four at diagonal direction. In the phosphorylation-dephosphorylation direction, two types of shuffling can produce the same results.

One shuffling contains two steps. First, one $\mathrm{C}_s^t$ loses one monomer, say $m_{00}$, and its probability is $L_{st}^{00} = \dfrac{\sum_m D_{\alpha_m \beta_m \gamma_m \delta_m} \alpha_m / (\alpha_m + \beta_m + \gamma_m + \delta_m)}{\sum_m D_{\alpha_m \beta_m \gamma_m \delta_m}}$. The other step is to receive one monomer from the pool that contains the four types of monomer contributed by all the other KaiC hexamers (as donors). For instance, the fraction of $m_{10}$ in the pool can be described by $R_{10} = \sum_{s,t} L_{st}^{10}$, where *s*, *t* exclude the receiver hexamer. Therefore, the reaction flow for $\mathrm{C}_s^t - \mathrm{m}_{00} + \mathrm{m}_{10}$ is $k_s L_{st}^{00} R_{10} [\mathrm{C}_s^t]$. Likewise, all the monomer shuffling process in Fig. S14A can be quantitatively calculated. We found that with the increase in the shuffle rate $k_s$, the period of KaiC phosphorylation becomes longer but the amplitude declines slightly, as shown in Fig. S14B.

## 2.10 Dynamic phase shifts by transient variations in KaiA concentration

We further explore the phase response curves of the *in vitro* Kai oscillator, using

the transient (4 hours) changes in KaiA concentrations as the stimulus pulses, i.e. 1/3×, 2/3×, 1.5× and 3× standard amount of KaiA are applied during the pulse. The raw dynamics under the stimuli are shown in the following figures.

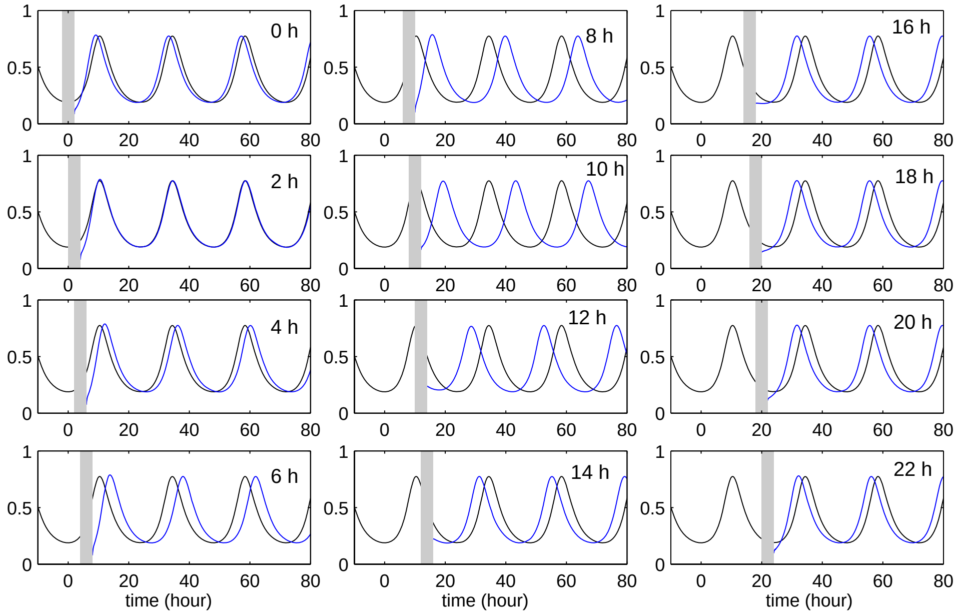


Fig. S15. KaiC phosphorylation dynamics perturbed by stimulus pulses of 1/3× standard KaiA concentration at different circadian time points. The black curves are the unperturbed dynamic profiles, while the blue curves are the profiles after stimulus pulses. The grey bar stands for one pulse with 4-hour duration.

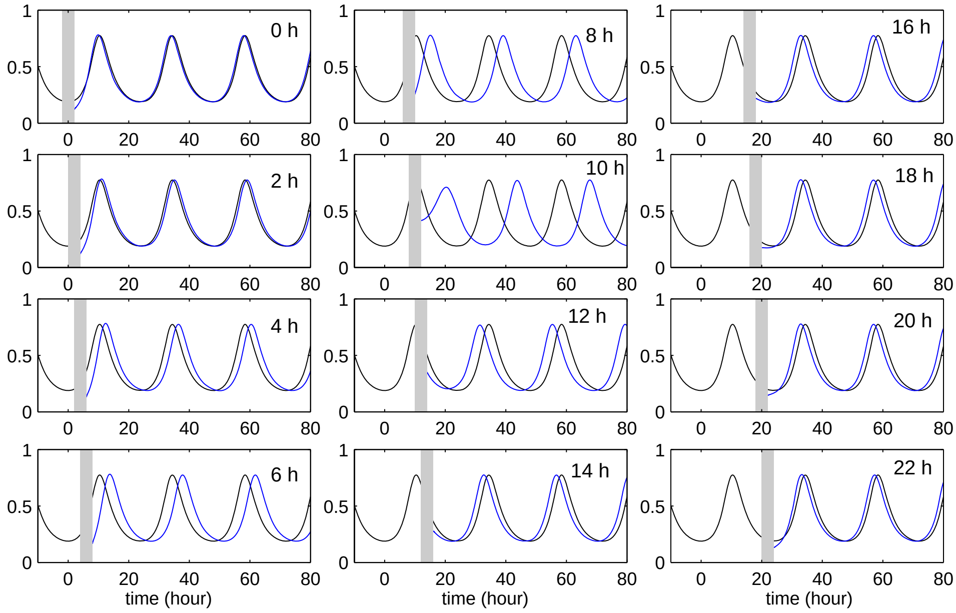


Fig. S16. KaiC phosphorylation dynamics perturbed by stimulus pulses of 2/3× standard KaiA

concentration at different circadian time points. The black curves are the unperturbed dynamic profiles, while the blue curves are the profiles after stimulus pulses. The grey bar stands for one pulse with 4-hour duration.

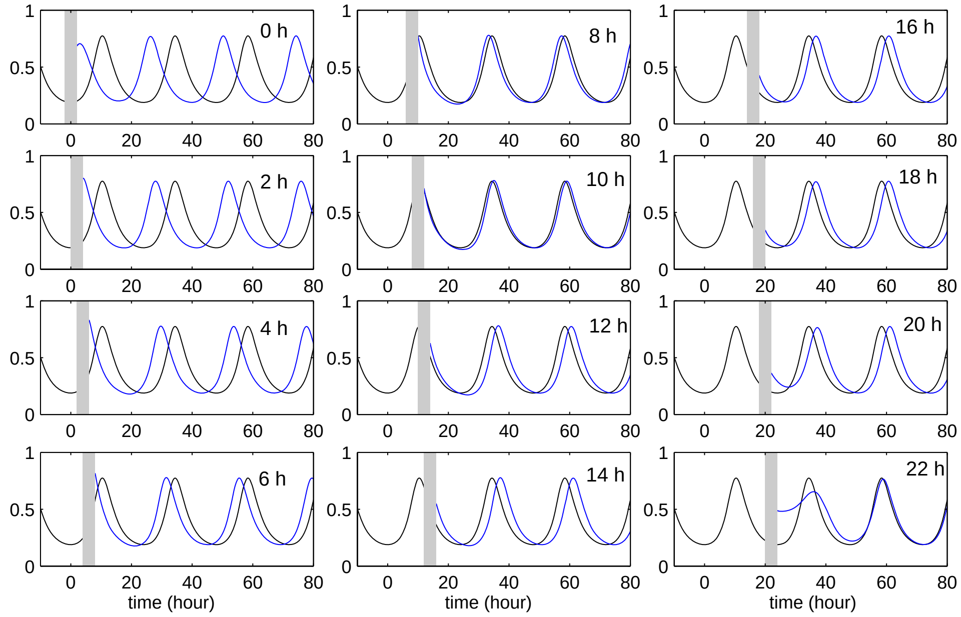


Fig. S17. KaiC phosphorylation dynamics perturbed by stimulus pulses of 1.5× standard KaiA concentration at different circadian time points. The black curves are the unperturbed dynamic profiles, while the blue curves are the profiles after stimulus pulses. The grey bar stands for one pulse with 4-hour duration.

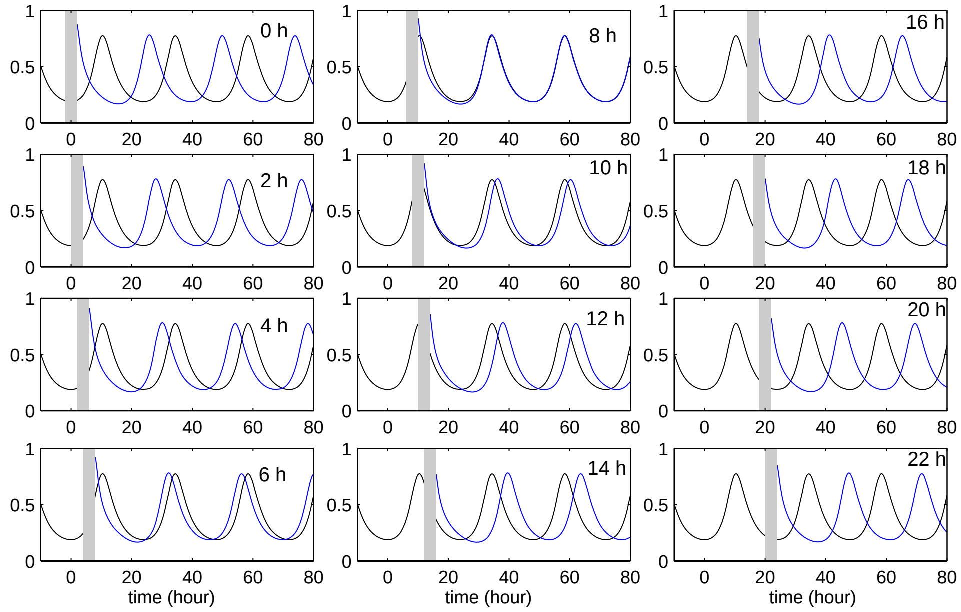


Fig. S18. KaiC phosphorylation dynamics perturbed by stimulus pulses of 3× standard KaiA concentration at different circadian time points. The black curves are the unperturbed dynamic profiles, while the blue curves are the profiles after stimulus pulses. The grey bar stands for one pulse with 4-hour duration.

# 3. References


1. Kageyama H, Nishiwaki T, Nakajima M, Iwasaki H, Oyama T, et al. (2006) Cyanobacterial circadian pacemaker: Kai protein complex dynamics in the KaiC phosphorylation cycle in vitro. Mol Cell 23: 161-171.
2. Winfree AT (1980) The Geometry of Biological Time. New York: Springer.
3. Kuramoto Y (1984) Chemical Oscillations, waves, and Turbulence. New York: Springer.
4. Micheletti C, Lattanzi G, Maritan A (2002) Elastic properties of proteins: insight on the folding process and evolutionary selection of native structures. J Mol Biol 321: 909-921.
5. Hawkins RJ, McLeish TC (2006) Coupling of global and local vibrational modes in dynamic allostery of proteins. Biophys J 91: 2055-2062.
6. Hawkins RJ, McLeish TC (2004) Coarse-grained model of entropic allostery. Phys Rev Lett 93: 098104.
7. Brooks B, Karplus M (1985) Normal modes for specific motions of macromolecules: application to the hinge-bending mode of lysozyme. Proc Natl Acad Sci U S A 82: 4995-4999.
8. Levitt M, Sander C, Stern PS (1985) Protein normal-mode dynamics: trypsin inhibitor, crambin, ribonuclease and lysozyme. J Mol Biol 181: 423-447.
9. Hayashi F, Iwase R, Uzumaki T, Ishiura M (2006) Hexamerization by the N-terminal domain and intersubunit phosphorylation by the C-terminal domain of cyanobacterial circadian clock protein KaiC. Biochem Biophys Res Commun 348: 864-872.
10. Nishiwaki T, Satomi Y, Kitayama Y, Terauchi K, Kiyohara R, et al. (2007) A sequential program of dual phosphorylation of KaiC as a basis for circadian rhythm in cyanobacteria. Embo J 26: 4029-4037.
11. Rust MJ, Markson JS, Lane WS, Fisher DS, O'Shea EK (2007) Ordered phosphorylation governs oscillation of a three-protein circadian clock. Science 318: 809-812.
12. Nishiwaki T, Satomi Y, Nakajima M, Lee C, Kiyohara R, et al. (2004) Role of KaiC phosphorylation in the circadian clock system of Synechococcus elongatus PCC 7942. Proc Natl Acad Sci U S A 101: 13927-13932.
13. Gillespie DT (1977) Exact stochastic simulation of coupled chemical reactions. J phys Chem 81: 2340-2361.
14. Tomita J, Nakajima M, Kondo T, Iwasaki H (2005) No transcription-translation feedback in circadian rhythm of KaiC phosphorylation. Science 307: 251-254.
15. Nakajima M, Imai K, Ito H, Nishiwaki T, Murayama Y, et al. (2005) Reconstitution of circadian oscillation of cyanobacterial KaiC phosphorylation in vitro. Science 308: 414-415.
16. van Zon JS, Lubensky DK, Altena PR, ten Wolde PR (2007) An allosteric model of circadian KaiC phosphorylation. Proc Natl Acad Sci U S A 104: 7420-7425.
17. Ermentrout B (2002) Simulating, Analyzing, and Animating Dynamical Systems: Society for Industrial and Applied Mathematics, Philadelphia.
