# Supplementary material for: Circadian KaiC Phosphorylation: A Multi-Layer Network
Source: PLoS Comput Biol. 2009 Nov 20;5(11):e1000568. doi: 10.1371/journal.pcbi.1000568 (PMC2773046; doi:10.1371/journal.pcbi.1000568)
Supplement: Table S1 — Expressions and descriptions for reaction flows. (0.02 MB PDF) [file pcbi.1000568.s002.pdf]

Table S1: Expressions and descriptions for reaction flows (J)

flow	expression	explanation
$Japt_s^t$	$kapt_s^t[AC_s^t]$	KaiAC (AC_s^t) phosphorylation at T432
$Japs_s^t$	$kaps_s^t[AC_s^t]$	KaiAC (AC_s^t) phosphorylation at S431
$Jdpt_s^t$	$kdpt_s^t[C_s^t]$	KaiC (C_s^t) dephosphorylation at T432
$Jdps_s^t$	$kdps_s^t[C_s^t]$	KaiC (C_s^t) dephosphorylation at S431
$Jbdpt_s^t$	$kbdpt_s^t[B_2C_s^t]$	KaiBC ($B_2C_s^t$) dephosphorylation at T432
$Jbdps_s^t$	$kbdps_s^t[B_2C_s^t]$	KaiBC ($B_2C_s^t$) dephosphorylation at S431
$Jabpt_s^t$	$kabpt_s^t([AB_2C_s^t]+[A_2B_2C_s^t]+[A_3B_2C_s^t])$	KaiABC ($AB_2C_s^t, A_2B_2C_s^t$ and $A_3B_2C_s^t$) phosphorylation at T432
$Jabps_s^t$	$kabps_s^t([AB_2C_s^t]+[A_2B_2C_s^t]+[A_3B_2C_s^t])$	KaiABC ($AB_2C_s^t$, $A_2B_2C_s^t$ and $A_3B_2C_s^t$) phosphorylation at S431
$Jabdpt_s^t$	$kabdpt_s^t([AB_2C_s^t]+[A_2B_2C_s^t]+[A_3B_2C_s^t])$	KaiABC ($AB_2C_s^t$, $A_2B_2C_s^t$ and $A_3B_2C_s^t$) dephosphorylation at T432
$Jabdps_s^t$	$kabdps_s^t([AB_2C_s^t]+[A_2B_2C_s^t]+[A_3B_2C_s^t])$	KaiABC ($AB_2C_s^t, A_2B_2C_s^t$ and $A_3B_2C_s^t$) dephosphorylation at S431

Descriptions of rate constants for KaiC (and Kai complexes) phosphorylation and dephosphorylation

rate constants	description
$kapt_s^t$	KaiAC (AC_s^t) phosphorylation at T432
$kaps_s^t$	KaiAC (AC_s^t) phosphorylation at S431

$kdpt_s^t$	KaiC (C_s^t) dephosphorylation at T432
$kdps_s^t$	KaiC (C_s^t) dephosphorylation at S431
$kbdpt_s^t$	KaiBC ($B_2C_s^t$) dephosphorylation at T432
$kbdps_s^t$	KaiBC ($B_2C_s^t$) dephosphorylation at S431
$kabpt_s^t$	KaiABC ($AB_2C_s^t$, $A_2B_2C_s^t$ and $A_3B_2C_s^t$) phosphorylation at T432
$kabps_s^t$	KaiABC ($AB_2C_s^t$, $A_2B_2C_s^t$ and $A_3B_2C_s^t$) phosphorylation at S431
$kabdpt_s^t$	KaiABC ($AB_2C_s^t$, $A_2B_2C_s^t$ and $A_3B_2C_s^t$) dephosphorylation at T432
$kabdps_s^t$	KaiABC ($AB_2C_s^t$, $A_2B_2C_s^t$ and $A_3B_2C_s^t$) dephosphorylation at S431
