# Supplementary material for: Circadian KaiC Phosphorylation: A Multi-Layer Network
Source: PLoS Comput Biol. 2009 Nov 20;5(11):e1000568. doi: 10.1371/journal.pcbi.1000568 (PMC2773046; doi:10.1371/journal.pcbi.1000568)
Supplement: Table S2 — Relationship between ST and subunit representations. (0.08 MB PDF) [file pcbi.1000568.s003.pdf]

Table S2: Relationship between *ST* and *subunit* representations based on *D* factor.

ST representation		*subunit representation*				*D* factor
T432	S431	m_{00}	m_{01}	m_{10}	m_{11}	*D*
0	0	6	0	0	0	1
1	0	5	0	1	0	6
2	0	4	0	2	0	15
3	0	3	0	3	0	20
4	0	2	0	4	0	15
5	0	1	0	5	0	6
6	0	0	0	6	0	1
0	1	5	1	0	0	6
1	1	4	1	1	0	30
		5	0	0	1	6
2	1	3	1	2	0	60
		4	0	1	1	30
3	1	2	1	3	0	60
		3	0	2	1	60
4	1	1	1	4	0	30
		2	0	3	1	60
5	1	0	1	5	0	6
		1	0	4	1	30
6	1	0	0	5	1	6

0	2	4	2	0	0	15
1	2	3	2	1	0	60
		4	1	0	1	30
2	2	2	2	2	0	90
		3	1	1	1	120
		4	0	0	2	15
3	2	1	2	3	0	60
		2	1	2	1	180
		3	0	1	2	60
4	2	0	2	4	0	15
		1	1	3	1	120
		2	0	2	2	90
5	2	0	1	4	1	30
		1	0	3	2	60
6	2	0	0	4	2	15
0	3	3	3	0	0	20
1	3	2	3	1	0	60
		3	2	0	1	60
2	3	1	3	2	0	60
		2	2	1	1	180
		3	1	0	2	60
3	3	0	3	3	0	20

		1	2	2	1	180
		2	1	1	2	180
		3	0	0	3	20
4	3	0	2	3	1	60
		1	1	2	2	180
		2	0	1	3	60
5	3	0	1	3	2	60
		1	0	2	3	60
6	3	0	0	3	3	20
0	4	2	4	0	0	15
1	4	1	4	1	0	30
		2	3	0	1	60
2	4	0	4	2	0	15
		1	3	1	1	120
		2	2	0	2	90
3	4	0	3	2	1	60
		1	2	1	2	180
		2	1	0	3	60
4	4	0	2	2	2	90
		1	1	1	3	120
		2	0	0	4	15
5	4	0	1	2	3	60

		1	0	1	4	30
6	4	0	0	2	4	15
0	5	1	5	0	0	6
1	5	0	5	1	0	6
		1	4	0	1	30
2	5	0	4	1	1	30
		1	3	0	2	60
3	5	0	3	1	2	60
		1	2	0	3	60
4	5	0	2	1	3	60
		1	1	0	4	30
5	5	0	1	1	4	30
		1	0	0	5	6
6	5	0	0	1	5	6
0	6	0	6	0	0	1
1	6	0	5	0	1	6
2	6	0	4	0	2	15
3	6	0	3	0	3	20
4	6	0	2	0	4	15
5	6	0	1	0	5	6
6	6	0	0	0	6	1
