# Supplementary material for: Circadian KaiC Phosphorylation: A Multi-Layer Network
Source: PLoS Comput Biol. 2009 Nov 20;5(11):e1000568. doi: 10.1371/journal.pcbi.1000568 (PMC2773046; doi:10.1371/journal.pcbi.1000568)
Supplement: Table S3 — Combinational factors of the triplets in KaiC hexamer. (0.05 MB PDF) [file pcbi.1000568.s004.pdf]

Table S3: Combinational factors of the triplets in KaiC hexamer

Triplets centered by the transition state interface	N factor
m_{00}-$\mathbf{m_{00}}$-m_{00}	$f(\alpha,1)f(\alpha-1,2)$
m_{00}-$\mathbf{m_{00}}$-m_{01}	$f(\alpha,1)f(\alpha-1,1)f(\beta,1)$
m_{00}-$\mathbf{m_{00}}$-m_{10}	$f(\alpha,1)f(\alpha-1,1)f(\gamma,1)$
m_{00}-$\mathbf{m_{00}}$-m_{11}	$f(\alpha,1)f(\alpha-1,1)f(\delta,1)$
m_{01}-$\mathbf{m_{00}}$-m_{01}	$f(\alpha,1)f(\beta,2)$
m_{01}-$\mathbf{m_{00}}$-m_{10}	$f(\alpha,1)f(\beta,1)f(\gamma,1)$
m_{01}-$\mathbf{m_{00}}$-m_{11}	$f(\alpha,1)f(\beta,1)f(\delta,1)$
m_{10}-$\mathbf{m_{00}}$-m_{10}	$f(\alpha,1)f(\gamma,2)$
m_{10}-$\mathbf{m_{00}}$-m_{11}	$f(\alpha,1)f(\gamma,1)f(\delta,1)$
m_{11}-$\mathbf{m_{00}}$-m_{11}	$f(\alpha,1)f(\delta,2)$
m_{00}-$\mathbf{m_{01}}$-m_{00}	$f(\beta,1)f(\alpha,2)$
m_{00}-$\mathbf{m_{01}}$-m_{01}	$f(\beta,1)f(\alpha,1)f(\beta-1,1)$
m_{00}-$\mathbf{m_{01}}$-m_{10}	$f(\beta,1)f(\alpha,1)f(\gamma,1)$
m_{00}-$\mathbf{m_{01}}$-m_{11}	$f(\beta,1)f(\alpha,1)f(\delta,1)$
m_{01}-$\mathbf{m_{01}}$-m_{01}	$f(\beta,1)f(\beta-1,2)$
m_{01}-$\mathbf{m_{01}}$-m_{10}	$f(\beta,1)f(\beta-1,1)f(\gamma,1)$
m_{01}-$\mathbf{m_{01}}$-m_{11}	$f(\beta,1)f(\beta-1,1)f(\delta,1)$
m_{10}-$\mathbf{m_{01}}$-m_{10}	$f(\beta,1)f(\gamma,2)$

m_{10}-$\mathbf{m_{01}}$-m_{11}	$f(\beta,1)f(\gamma,1)f(\delta,1)$
m_{11}-$\mathbf{m_{01}}$-m_{11}	$f(\beta,1)f(\delta,2)$
m_{00}-$\mathbf{m_{10}}$-m_{00}	$f(\gamma,1)f(\alpha,2)$
m_{00}-$\mathbf{m_{10}}$-m_{01}	$f(\gamma,1)f(\alpha,1)f(\beta,1)$
m_{00}-$\mathbf{m_{10}}$-m_{10}	$f(\gamma,1)f(\alpha,1)f(\gamma-1,1)$
m_{00}-$\mathbf{m_{10}}$-m_{11}	$f(\gamma,1)f(\alpha,1)f(\delta,1)$
m_{01}-$\mathbf{m_{10}}$-m_{01}	$f(\gamma,1)f(\beta,2)$
m_{01}-$\mathbf{m_{10}}$-m_{10}	$f(\gamma,1)f(\beta,1)f(\gamma-1,1)$
m_{01}-$\mathbf{m_{10}}$-m_{11}	$f(\gamma,1)f(\beta,1)f(\delta,1)$
m_{10}-$\mathbf{m_{10}}$-m_{10}	$f(\gamma,1)f(\gamma-1,2)$
m_{10}-$\mathbf{m_{10}}$-m_{11}	$f(\gamma,1)f(\gamma-1,1)f(\delta,1)$
m_{11}-$\mathbf{m_{10}}$-m_{11}	$f(\gamma,1)f(\delta,2)$
m_{00}-$\mathbf{m_{11}}$-m_{00}	$f(\delta,1)f(\alpha,2)$
m_{00}-$\mathbf{m_{11}}$-m_{01}	$f(\delta,1)f(\alpha,1)f(\beta,1)$
m_{00}-$\mathbf{m_{11}}$-m_{10}	$f(\delta,1)f(\alpha,1)f(\gamma,1)$
m_{00}-$\mathbf{m_{11}}$-m_{11}	$f(\delta,1)f(\alpha,1)f(\delta-1,1)$
m_{01}-$\mathbf{m_{11}}$-m_{01}	$f(\delta,1)f(\beta,2)$
m_{01}-$\mathbf{m_{11}}$-m_{10}	$f(\delta,1)f(\beta,1)f(\gamma,1)$
m_{01}-$\mathbf{m_{11}}$-m_{11}	$f(\delta,1)f(\beta,1)f(\delta-1,1)$
m_{10}-$\mathbf{m_{11}}$-m_{10}	$f(\delta,1)f(\gamma,2)$

m_{10}-$\mathbf{m_{11}}$-m_{11}	$f(\delta,1)f(\gamma,1)f(\delta-1,1)$
m_{11}-$\mathbf{m_{11}}$-m_{11}	$f(\delta,1)f(\delta-1,2)$

It is defined: $f(n,k)=\begin{cases}\dfrac{n!}{k!(n-k)!} & \text{if } n\geq 1,\ 0<k\leq n \\ 0 & \text{else}\end{cases}$. α, β, γ and δ are the numbers of m_{00}, m_{01}, m_{10} and m_{11} in one KaiC hexamer, respectively. The m_{ij} marked bold is the reaction interface.
