# Supplementary material for: Circadian KaiC Phosphorylation: A Multi-Layer Network
Source: PLoS Comput Biol. 2009 Nov 20;5(11):e1000568. doi: 10.1371/journal.pcbi.1000568 (PMC2773046; doi:10.1371/journal.pcbi.1000568)
Supplement: Table S4 — Dissociation constants and rate constants in Kai reaction network. (0.20 MB PDF) [file pcbi.1000568.s005.pdf]

Table S4: Dissociation constants and rate constants in Kai reaction network. (The values in the round brackets are specific for simulations that consistent with experiments by Rust et al., and details can be found in section 2.6 in the Text S1.)

Dissociation constants for KaiA-KaiC binding-unbinding: Ka_s^t (μM)							
T432 \ S431	0	1	2	3	4	5	6
0	0.3009	0.3009	0.3009	0.3009	0.3009	0.3009	0.3009
1	0.3825	0.3825	0.3825	0.3825	0.3825	0.3825	0.3825
2	0.5616	0.5616	0.5616	0.5616	0.5616	0.5616	0.5616
3	1.0382	1.0382	1.0382	1.0382	1.0382	1.0382	1.0382
4	2.7747	2.7747	2.7747	2.7747	2.7747	2.7747	2.7747
5	13.3752	13.3752	13.3752	13.3752	13.3752	13.3752	13.3752
6	165.6677	165.6677	165.6677	165.6677	165.6677	165.6677	165.6677
Dissociation constants for KaiA-KaiBC binding-unbinding: Kba_s^t (μM)							
T432 \ S431	0	1	2	3	4	5	6
0	0.0003 (0.0001)	0.0008 (0.0005)	0.0028 (0.0017)	0.0091 (0.0058)	0.0298 (0.0198)	0.0890 (0.0585)	0.1656 (0.0755)
1	0.0011 (0.0007)	0.0031 (0.0020)	0.0086 (0.0056)	0.0234 (0.0160)	0.0616 (0.0431)	0.1419 (0.0941)	0.2316 (0.1183)
2	0.0050 (0.0034)	0.0116 (0.0080)	0.0263 (0.0188)	0.0585 (0.0428)	0.1218 (0.0887)	0.2217 (0.1515)	0.3220 (0.1856)
3	0.0225 (0.0167)	0.0425 (0.0323)	0.0787 (0.0608)	0.1388 (0.1076)	0.2276 (0.1730)	0.3384 (0.2438)	0.4450 (0.2911)
4	0.0980 (0.0802)	0.1487 (0.1231)	0.2157 (0.1787)	0.2992 (0.2454)	0.3969 (0.3190)	0.5034 (0.3923)	0.6105 (0.4565)

5	0.3720 (0.3366)	0.4254 (0.3817)	0.4864 (0.4329)	0.5561 (0.4909)	0.6359 (0.5567)	0.7271 (0.6313)	0.8314 (0.7159)
6	0.6162	0.6810	0.7526	0.8318	0.9193	1.0160	1.1228

Rate constants for KaiB-KaiC association: kb_s^t (μM^{-2}h^{-1})

T432 / S431	0	1	2	3	4	5	6
0	0.000E+00	0.000E+00 (2.990E-01)	2.261E+00 (2.990E-01)	2.261E+00 (2.990E-01)	2.261E+00 (2.990E-01)	2.261E+00 (2.990E-01)	2.261E+00 (2.990E-01)
1	1.225E+01 (2.186E+00)	1.225E+01 (2.186E+00)	1.225E+01 (2.186E+00)	1.225E+01 (2.186E+00)	1.225E+01 (2.186E+00)	1.225E+01 (2.186E+00)	1.225E+01 (2.186E+00)
2	5.926E+01 (1.428E+01)	5.926E+01 (1.428E+01)	5.926E+01 (1.428E+01)	5.926E+01 (1.428E+01)	5.926E+01 (1.428E+01)	5.926E+01 (1.428E+01)	5.926E+01 (1.428E+01)
3	2.483E+02 (8.075E+01)	2.483E+02 (8.075E+01)	2.483E+02 (8.075E+01)	2.483E+02 (8.075E+01)	2.483E+02 (8.075E+01)	2.483E+02 (8.075E+01)	2.483E+02 (8.075E+01)
4	8.657E+02 (3.801E+02)	8.657E+02 (3.801E+02)	8.657E+02 (3.801E+02)	8.657E+02 (3.801E+02)	8.657E+02 (3.801E+02)	8.657E+02 (3.801E+02)	8.657E+02 (3.801E+02)
5	2.389E+03 (1.416E+03)	2.389E+03 (1.416E+03)	2.389E+03 (1.416E+03)	2.389E+03 (1.416E+03)	2.389E+03 (1.416E+03)	2.389E+03 (1.416E+03)	2.389E+03 (1.416E+03)
6	4.891E+03 (3.913E+03)	4.891E+03 (3.913E+03)	4.891E+03 (3.913E+03)	4.891E+03 (3.913E+03)	4.891E+03 (3.913E+03)	4.891E+03 (3.913E+03)	4.891E+03 (3.913E+03)

Rate constants for KaiB-KaiC dissociation: kdb_s^t (h^{-1})

T432 / S431	0	1	2	3	4	5	6
0	90	90	90	90	90	90	90
1	90	90	90	90	90	90	90
2	90	90	90	90	90	90	90
3	90	90	90	90	90	90	90
4	90	90	90	90	90	90	90

5	90	90	90	90	90	90	90
6	90	90	90	90	90	90	90
Rate constants for KaiAC phosphorylation at T432: $kapt_s^t$ (h^{-1})							
T432 \ S431	0	1	2	3	4	5	6
0	7.6771 (7.5786)	7.6771 (7.5786)	7.6771 (7.5786)	7.6771 (7.5786)	7.6771 (7.5786)	7.6771 (7.5786)	---
1	7.6771 (7.5786)	7.6771 (7.5786)	7.6771 (7.5786)	7.6771 (7.5786)	7.6771 (7.5786)	7.6771 (7.5786)	---
2	7.6771 (7.5786)	7.6771 (7.5786)	7.6771 (7.5786)	7.6771 (7.5786)	7.6771 (7.5786)	7.6771 (7.5786)	---
3	7.6771 (7.5786)	7.6771 (7.5786)	7.6771 (7.5786)	7.6771 (7.5786)	7.6771 (7.5786)	7.6771 (7.5786)	---
4	7.6771 (7.5786)	7.6771 (7.5786)	7.6771 (7.5786)	7.6771 (7.5786)	7.6771 (7.5786)	7.6771 (7.5786)	---
5	7.6771 (7.5786)	7.6771 (7.5786)	7.6771 (7.5786)	7.6771 (7.5786)	7.6771 (7.5786)	7.6771 (7.5786)	---
6	7.6771 (7.5786)	7.6771 (7.5786)	7.6771 (7.5786)	7.6771 (7.5786)	7.6771 (7.5786)	7.6771 (7.5786)	---
Rate constants for KaiAC phosphorylation at S431: $kaps_s^t$ (h^{-1})							
T432 \ S431	0	1	2	3	4	5	6
0	0.0378 (0.0129)	0.0671 (0.0288)	0.1194 (0.0640)	0.2124 (0.1423)	0.3777 (0.3163)	0.6717 (0.7032)	1.1947 (1.5635)
1	0.0378 (0.0129)	0.0671 (0.0288)	0.1194 (0.0640)	0.2124 (0.1423)	0.3777 (0.3163)	0.6717 (0.7032)	1.1947 (1.5635)
2	0.0378 (0.0129)	0.0671 (0.0288)	0.1194 (0.0640)	0.2124 (0.1423)	0.3777 (0.3163)	0.6717 (0.7032)	1.1947 (1.5635)
3	0.0378 (0.0129)	0.0671 (0.0288)	0.1194 (0.0640)	0.2124 (0.1423)	0.3777 (0.3163)	0.6717 (0.7032)	1.1947 (1.5635)
4	0.0378 (0.0129)	0.0671 (0.0288)	0.1194 (0.0640)	0.2124 (0.1423)	0.3777 (0.3163)	0.6717 (0.7032)	1.1947 (1.5635)

5	0.0378 (0.0129)	0.0671 (0.0288)	0.1194 (0.0640)	0.2124 (0.1423)	0.3777 (0.3163)	0.6717 (0.7032)	1.1947 (1.5635)
6	---	---	---	---	---	---	---

Rate constants for KaiABC phosphorylation at T432: $kabpt_s^t$ (h^{-1})

T432 \ S431	0	1	2	3	4	5	6
0	1.0876 (1.1200)	1.7927 (1.8506)	3.0494 (3.1561)	5.3665 (5.5696)	9.7982 (10.1988)	18.6165 (19.4382)	---
1	0.6574 (0.7032)	1.1007 (1.1830)	1.9089 (2.0623)	3.4394 (3.7362)	6.4600 (7.0586)	12.6958 (13.9595)	---
2	0.4099 (0.4533)	0.6945 (0.7739)	1.2235 (1.3742)	2.2487 (2.5472)	4.3294 (4.9493)	8.7719 (10.1271)	---
3	0.2630 (0.2995)	0.4496 (0.5173)	0.8018 (0.9328)	1.4982 (1.7637)	2.9473 (3.5140)	6.1374 (7.4191)	---
4	0.1733 (0.2025)	0.2981 (0.3529)	0.5366 (0.6444)	1.0164 (1.2393)	2.0368 (2.5251)	4.3464 (5.4868)	---
5	0.1171 (0.1399)	0.2021 (0.2454)	0.3664 (0.4527)	0.7014 (0.8832)	1.4279 (1.8355)	3.1141 (4.0950)	---
6	0.0810 (0.0987)	0.1400 (0.1738)	0.2549 (0.3230)	0.4920 (0.6380)	1.0149 (1.3491)	2.2563 (3.0832)	---

Rate constants for KaiABC phosphorylation at S431: $kabps_s^t$ (h^{-1})

T432 \ S431	0	1	2	3	4	5	6
0	0.0208 (0.0050)	0.0558 (0.0167)	0.1649 (0.0628)	0.5408 (0.2714)	1.9857 (1.3591)	8.2507 (8.0048)	39.2369 (56.3038)
1	0.0148 (0.0039)	0.0387 (0.0123)	0.1120 (0.0446)	0.3627 (0.1868)	1.3271 (0.9184)	5.5560 (5.3834)	26.9805 (38.3157)
2	0.0107 (0.0030)	0.0274 (0.0092)	0.0776 (0.0322)	0.2478 (0.1309)	0.9018 (0.6308)	3.7951 (3.6728)	18.7696 (26.3878)
3	0.0080 (0.0024)	0.0198 (0.0070)	0.0548 (0.0236)	0.1724 (0.0932)	0.6227 (0.4401)	2.6278 (2.5403)	13.2042 (18.3830)
4	0.0060 (0.0019)	0.0145 (0.0054)	0.0394 (0.0176)	0.1219 (0.0675)	0.4365 (0.3116)	1.8434 (1.7802)	9.3894 (12.9488)

5	0.0046<br>(0.0016)	0.0109<br>(0.0043)	0.0288<br>(0.0134)	0.0876<br>(0.0496)	0.3104<br>(0.2238)	1.3094<br>(1.2633)	6.7462<br>(9.2185)
6	---	---	---	---	---	---	---
Rate constants for free KaiC dephosphorylation at T432: $kdpt_s^t$ (h^{-1})							
T432 \ S431	0	1	2	3	4	5	6
0	---	1.1575<br>(1.3402)	1.1575<br>(1.3402)	1.1575<br>(1.3402)	1.1575<br>(1.3402)	1.1575<br>(1.3402)	1.1575<br>(1.3402)
1	---	1.5466<br>(1.8364)	1.4995<br>(1.7832)	1.4540<br>(1.7316)	1.4098<br>(1.6814)	1.3669<br>(1.6327)	1.3254<br>(1.5854)
2	---	2.0965<br>(2.5667)	1.9645<br>(2.4112)	1.8416<br>(2.2661)	1.7273<br>(2.1307)	1.6207<br>(2.0042)	1.5214<br>(1.8860)
3	---	2.8861<br>(3.6648)	2.6041<br>(3.3170)	2.3531<br>(3.0064)	2.1293<br>(2.7286)	1.9296<br>(2.4799)	1.7510<br>(2.2568)
4	---	4.0383<br>(5.3548)	3.4949<br>(4.6479)	3.0339<br>(4.0465)	2.6417<br>(3.5334)	2.3070<br>(3.0941)	2.0205<br>(2.7171)
5	---	5.7494<br>(8.0216)	4.7516<br>(6.6429)	3.9486<br>(5.5310)	3.2988<br>(4.6294)	2.7701<br>(3.8945)	2.3377<br>(3.2922)
6	---	8.3372<br>(12.3453)	6.5489<br>(9.6973)	5.1897<br>(7.6846)	4.1473<br>(6.1411)	3.3409<br>(4.9470)	2.7119<br>(4.0157)
Rate constants for free KaiC dephosphorylation at S431: $kdps_s^t$ (h^{-1})							
T432 \ S431	0	1	2	3	4	5	6
0	---	---	---	---	---	---	---
1	0.2204<br>(0.2175)	0.2204<br>(0.2175)	0.2204<br>(0.2175)	0.2204<br>(0.2175)	0.2204<br>(0.2175)	0.2204<br>(0.2175)	0.2204<br>(0.2175)
2	0.3602<br>(0.3556)	0.3467<br>(0.3422)	0.3337<br>(0.3294)	0.3212<br>(0.3171)	0.3091<br>(0.3052)	0.2975<br>(0.2937)	0.2864<br>(0.2827)
3	0.6120<br>(0.6041)	0.5635<br>(0.5563)	0.5192<br>(0.5126)	0.4787<br>(0.4725)	0.4415<br>(0.4359)	0.4075<br>(0.4023)	0.3763<br>(0.3715)
4	1.0850<br>(1.0711)	0.9493<br>(0.9371)	0.8321<br>(0.8214)	0.7307<br>(0.7213)	0.6429<br>(0.6346)	0.5666<br>(0.5593)	0.5002<br>(0.4938)

5	2.0168 (1.9909)	1.6630 (1.6417)	1.3770 (1.3593)	1.1447 (1.1300)	0.9554 (0.9431)	0.8005 (0.7902)	0.6732 (0.6645)
6	3.9515 (3.9008)	3.0421 (3.0030)	2.3599 (2.3296)	1.8443 (1.8206)	1.4516 (1.4329)	1.1503 (1.1356)	0.9177 (0.9059)

Rate constants for KaiBC dephosphorylation at T432: $kbdpt_s^t$ (h^{-1})

T432 \ S431	0	1	2	3	4	5	6
0	---	0.8483 (1.4720)	0.8483 (1.4720)	0.8483 (1.4720)	0.8483 (1.4720)	0.8483 (1.4720)	0.8483 (1.4720)
1	---	1.3064 (2.1487)	1.2603 (2.0651)	1.2159 (1.9847)	1.1730 (1.9075)	1.1316 (1.8333)	1.0917 (1.7619)
2	---	2.0880 (3.2128)	1.9313 (2.9521)	1.7875 (2.7145)	1.6555 (2.4977)	1.5342 (2.2999)	1.4226 (2.1192)
3	---	3.4766 (4.9294)	3.0613 (4.3052)	2.7012 (3.7685)	2.3885 (3.3062)	2.1162 (2.9069)	1.8788 (2.5615)
4	---	6.0563 (7.7752)	5.0350 (6.4126)	4.2054 (5.3148)	3.5283 (4.4259)	2.9733 (3.7027)	2.5164 (3.1117)
5	---	11.0899 (12.6324)	8.6226 (9.7681)	6.7617 (7.6199)	5.3461 (5.9944)	4.2604 (4.7540)	3.4210 (3.7997)
6	---	21.4593 (21.1839)	15.4340 (15.2359)	11.2590 (11.1145)	8.3245 (8.2176)	6.2337 (6.1537)	4.7248 (4.6642)

Rate constants for KaiBC dephosphorylation at S431: $kbdps_s^t$ (h^{-1})

T432 \ S431	0	1	2	3	4	5	6
0	---	---	---	---	---	---	---
1	0.3472 (0.3427)	0.2384 (0.2044)	0.1637 (0.1219)	0.1124 (0.0727)	0.0772 (0.0433)	0.0530 (0.0258)	0.0364 (0.0154)
2	0.6072 (0.5994)	0.3864 (0.3273)	0.2474 (0.1802)	0.1594 (0.1001)	0.1033 (0.0560)	0.0674 (0.0316)	0.0442 (0.0180)
3	1.1193 (1.1050)	0.6529 (0.5457)	0.3862 (0.2747)	0.2317 (0.1409)	0.1408 (0.0736)	0.0868 (0.0391)	0.0542 (0.0212)
4	2.1893 (2.1612)	1.1552 (0.9516)	0.6246 (0.4328)	0.3458 (0.2032)	0.1959 (0.0983)	0.1135 (0.0490)	0.0672 (0.0252)

5	4.5763 (4.5175)	2.1512 (1.7437)	1.0500 (0.7073)	0.5314 (0.3009)	0.2783 (0.1339)	0.1507 (0.0622)	0.0842 (0.0301)
6	10.3096 (10.1773)	4.2403 (3.3771)	1.8423 (1.2034)	0.8428 (0.4585)	0.4047 (0.1861)	0.2034 (0.0801)	0.1067 (0.0365)

Rate constants for KaiABC dephosphorylation at T432: $kabdpt_s^t$ (h^{-1})

T432 \ S431	0	1	2	3	4	5	6
0	---	0.0497 (0.0082)	0.0423 (0.0070)	0.0363 (0.0060)	0.0314 (0.0052)	0.0274 (0.0045)	0.0241 (0.0040)
1	---	0.1080 (0.0178)	0.0865 (0.0142)	0.0703 (0.0116)	0.0578 (0.0095)	0.0482 (0.0079)	0.0405 (0.0067)
2	---	0.2561 (0.0421)	0.1903 (0.0313)	0.1443 (0.0237)	0.1115 (0.0183)	0.0876 (0.0144)	0.0699 (0.0115)
3	---	0.6683 (0.1100)	0.4522 (0.0744)	0.3150 (0.0518)	0.2254 (0.0371)	0.1652 (0.0272)	0.1237 (0.0204)
4	---	1.9390 (0.3190)	1.1693 (0.1924)	0.7350 (0.1209)	0.4796 (0.0789)	0.3236 (0.0532)	0.2251 (0.0370)
5	---	6.3228 (1.0403)	3.3147 (0.5454)	1.8417 (0.3030)	1.0774 (0.1773)	0.6600 (0.1086)	0.4213 (0.0693)
6	---	23.4598 (3.8598)	10.3869 (1.7089)	4.9824 (0.8197)	2.5643 (0.4219)	1.4043 (0.2310)	0.8124 (0.1337)

Rate constants for KaiABC dephosphorylation at S431: $kabdps_s^t$ (h^{-1})

T432 \ S431	0	1	2	3	4	5	6
0	---	---	---	---	---	---	---
1	0.0230 (0.0273)	0.0121 (0.0143)	0.0067 (0.0079)	0.0039 (0.0046)	0.0023 (0.0028)	0.0015 (0.0017)	0.0010 (0.0011)
2	0.0342 (0.0405)	0.0166 (0.0196)	0.0086 (0.0101)	0.0047 (0.0055)	0.0027 (0.0032)	0.0016 (0.0019)	0.0010 (0.0012)
3	0.0523 (0.0619)	0.0232 (0.0275)	0.0111 (0.0132)	0.0057 (0.0068)	0.0031 (0.0037)	0.0018 (0.0022)	0.0011 (0.0013)
4	0.0823 (0.0975)	0.0331 (0.0392)	0.0147 (0.0174)	0.0071 (0.0084)	0.0037 (0.0044)	0.0021 (0.0024)	0.0012 (0.0014)

5	0.1340<br>(0.1587)	0.0484<br>(0.0574)	0.0197<br>(0.0233)	0.0088<br>(0.0105)	0.0043<br>(0.0052)	0.0023<br>(0.0027)	0.0013<br>(0.0016)
6	0.2259<br>(0.2677)	0.0725<br>(0.0859)	0.0267<br>(0.0317)	0.0111<br>(0.0132)	0.0052<br>(0.0061)	0.0026<br>(0.0031)	0.0015<br>(0.0017)
