# Supplementary material for: Circadian KaiC Phosphorylation: A Multi-Layer Network
Source: PLoS Comput Biol. 2009 Nov 20;5(11):e1000568. doi: 10.1371/journal.pcbi.1000568 (PMC2773046; doi:10.1371/journal.pcbi.1000568)
Supplement: Table S5 — Parameters in the estimation of free energies of all reaction and interaction processes in Kai network. (0.12 MB PDF) [file pcbi.1000568.s006.pdf]

Table S5: Parameters in the estimation of free energies of all reaction and interaction processes in Kai network.

Parameters in the estimation of free energies of activation in KaiC (and its complexes) phosphorylation and dephosphorylation				
	free KaiC	KaiAC	KaiBC	KaiABC
$\Delta G_{\mathrm{p}}^{\neq *}$ ($k_{\mathrm{B}}T$)	25	25	25	25
$\Delta G_{\mathrm{d}}^{\neq *}$ ($k_{\mathrm{B}}T$)	20	20	20	20
$\Delta\Delta E_{\mathrm{p}}^{\neq}$ ($k_{\mathrm{B}}T$)	----	-13.5	----	-11.4
$\Delta\Delta E_{\mathrm{d}}^{\neq}$ ($k_{\mathrm{B}}T$)	-10.6	----	-10	-10.6
$\sigma_{\mathrm{00Tp}}^{\neq}$	----	0.3704	----	0.5412 (0.5439)
$\sigma_{\mathrm{01Tp}}^{\neq}$	----	0.3704	----	0.5088 (0.5263)
$\sigma_{\mathrm{00Sp}}^{\neq}$	----	0.2778 (0.2222)	----	0.3684 (0.2807)
$\sigma_{\mathrm{10Sp}}^{\neq}$	----	0.4593 (0.4741)	----	0.6316 (0.6491)
$\sigma_{\mathrm{10Tdp}}^{\neq}$	0.3962 (0.3774)	----	0.38 (0.42)	0.2075
$\sigma_{\mathrm{11Tdp}}^{\neq}$	0.3774	----	0.4	0.4528
$\sigma_{\mathrm{01Sdp}}^{\neq}$	0.4906	----	0.46	0.4151
$\sigma_{\mathrm{11Sdp}}^{\neq}$	0.4906	----	0.3 (0.24)	0.2642

λ_{00}	1	1	1	1
λ_{01}	0.8696	1	0.8696	0.9
λ_{10}	1	1	1	1.1
λ_{11}	0.9259	1	0.9259	1.05
ω	0.23	0.41	0.41	0.41
Apt_0 (h^{-1})	----	657.3658 (648.9298)	----	146.0928 (144.2179)
Aps_0 (h^{-1})	----	18.8379 (18.5961)	----	44.8530 (44.2774)
$Adpt_0$ (h^{-1})	79.8605 (118.2535)	----	88.0171 (86.8875)	27.0190 (4.4454)
$Adps_0$ (h^{-1})	4.4438 (4.3868)	----	11.6599 (11.5103)	0.5629 (0.6668)

Parameters in estimation of free energies for association-dissociation of Kai proteins

	KaiA-KaiC	KaiB-KaiC	KaiA-KaiBC
ΔG_{00} ($k_B T$)	-1	-2.2 (-2.5)	4 (4.1)
ΔG_{01} ($k_B T$)	-1	-0.1	2.5
ΔG_{10} ($k_B T$)	-1	-2.2 (-2.5)	2.8 (2.85)
ΔG_{11} ($k_B T$)	-1	-0.1	2.4
h_{00}	1.0	1.2	0.2
h_{01}	1.6	1.53	1.2

<table>
<tr><td>h_{10}</td><td>1.0</td><td colspan="2">1.2</td><td>1.1 (1.2)</td></tr>
<tr><td>h_{11}</td><td>1.6</td><td colspan="2">1.53</td><td>1.2</td></tr>
<tr><td>Ψ ($k_B T$)</td><td>-0.4</td><td colspan="2">-0.5</td><td>0.4</td></tr>
<tr><td rowspan="2">K_0 (μM)</td><td rowspan="2">5e-4</td><td>kb_0 (μ$M^{-2}h^{-1}$)</td><td>5.4e6 (4.4e6)</td><td rowspan="2">6.65e6</td></tr>
<tr><td>kdb_0 (h^{-1})</td><td>90</td></tr>
</table>

Apt_0, Aps_0, $Adpt_0$ and $Adps_0$ are the pre-exponential factors in Arrhenius equation for calculation of the rate constants for KaiC phosphorylation and dephosphorylation. K_0 is the pre-exponential factor for calculation of the dissociation constants. The values in the round brackets are specific for simulations that consistent with experiments by Rust et al. (details see section 2.6 in the Text S1).
